# Assessment of Feeding Intensity in Juvenile Asian Crucian Carp Using Binocular Vision and 2D–3D Spatial Feature Fusion

**DOI:** 10.3390/ani16213361

**Published:** 2026-10-24

**Authors:** Lu Zhang, Wenhui Ni, Zunxu Liu, Yue Li, Hao Yang, Shunshun Zhou

**Affiliations:** 1 College of Information and Artificial Intelligence, Yangzhou University, Yangzhou 225127, China; niwenhuiyzhh@163.com (W.N.); lzx_0126@163.com (Z.L.); y751923962@163.com (H.Y.); 15056721964@163.com (S.Z.); 2 Jiangsu Province Engineering Research Centre of Knowledge Management and Intelligent Service, Yangzhou 225127, China; 3 College of Information and Electrical Engineering, China Agricultural University, Beijing 100083, China; liyue2024@cau.edu.cn

**Keywords:** aquaculture, binocular vision, feeding intensity assessment, spatial features, feature extraction

## Abstract

Accurate assessment of fish feeding intensity is essential for understanding feeding demand and optimising aquaculture efficiency. Fish feeding behaviour is inherently a 3D spatial activity, yet monocular vision can only provide 2D image information, capturing feature variations on a single projection plane. This makes it incapable of effectively characterising the fish’s positional and behavioural features in 3D space, thereby limiting the accuracy of feeding intensity assessment. Therefore, a binocular vision-based assessment method for fish feeding intensity via 2D and 3D spatial feature fusion is proposed. Binocular fish images are acquired from a lateral perspective. Fish targets are then segmented using image processing techniques, and target feature points are extracted. On this basis, the 2D spatial features of the fish are derived. To address missing feature points caused by fish overlap and occlusion in binocular images, a disparity map-based Missing Point Restoration (MPR) method is proposed for feature restoration and matching. Furthermore, a Weighted Delaunay Triangulation (WDT) algorithm is proposed to ensure accurate 3D feature computation. Notably, the extracted 2D and 3D spatial features have clear physical meanings and can directly characterise feeding-related spatial behaviour, thereby improving the interpretability of feeding intensity assessment. Finally, a classification model based on Tabular Prior-data Fitted Network (TabPFN) is constructed for assessing fish feeding intensity. By extracting 3D spatial features via binocular vision, the proposed method overcomes the inadequacy of 2D features in characterising fish vertical behaviour, effectively compensates for depth-related assessment errors, and improves feeding intensity assessment accuracy. The proposed method was tested on a real fish feeding dataset, achieving an accuracy of 95.92%, precision of 95.97%, recall of 95.95%, specificity of 98.63%, and F1-Score of 95.96%, indicating its potential for fish feeding intensity assessment.

## Simple Summary

Accurately assessing fish feeding intensity is essential for optimising feeding management and reducing aquaculture costs. However, existing methods typically rely on single superficial features such as texture or behaviour, which struggle to capture the real 3D spatial changes in fish in the water, limiting assessment accuracy. To address this, we developed a new approach using binocular vision technology to more comprehensively record the spatial behaviour of fish. This method integrates both 2D and 3D spatial information and can effectively distinguish different levels of feeding intensity. Tested on a real-world fish feeding dataset, the method achieved an accuracy of 95.92%. This technology offers an intelligent feeding management solution for aquaculture, helping to save feed costs and promote sustainable fisheries development.

## 1. Introduction

In aquaculture, feed provides the essential energy required for the fish’s growth and reproduction. Consequently, how to achieve precise feeding has long been a topic of significant concern. Traditional aquaculture practices rely on manual observation and experience to assess fish feeding intensity and formulate feeding strategies. This approach is highly subjective, and can easily lead to underfeeding or overfeeding, affecting fish growth and development [1,2,3]. Therefore, accurate assessment of fish feeding intensity and the subsequent formulation of feeding strategies are significant for achieving precise feeding and improving aquaculture efficiency.

Computer vision, as a non-contact and non-invasive technique, has emerged as an important research tool for the intelligent development of aquaculture, owing to its ease of use and high efficiency [4,5,6]. Fish exhibit distinct behavioural patterns under different feeding intensities, allowing feeding intensity to be assessed through behavioural analysis using computer vision, thereby facilitating precision feeding. Currently, computer vision-based analysis of fish feeding behaviour can be broadly categorised into two categories: monocular vision-based and binocular vision-based methods.

Monocular vision-based methods primarily rely on 2D images to analyse fish behaviour. Some researchers have extracted texture features or behavioural features from monocular fish images to analyse fish behaviour and achieve feeding intensity assessment [7,8,9]. For example, Wu et al. [10] extracted fish body angle features by image processing techniques, including turning angle, tail curvature and angular velocity, and used the combined data of turning angle and tail curvature to assess changes in fish behavioural features. An improved kinetic energy model was employed to combine feature variations with angular velocity, thereby achieving the assessment of swimming intensity, and fish stress behaviour under hunger conditions was further identified through swimming intensity detection. Moreover, some researchers have employed deep learning methods to analyse fish behaviour and to assess feeding intensity [11,12,13]. For example, Feng et al. [14] proposed a multi-step image pre-enhancement strategy to address complex environmental conditions and employed the EfficientNet neural network model, thereby achieving the assessment of fish feeding intensity under complex illumination conditions. Zheng et al. [15] proposed a Spatio-Temporal Attention Network (STAN) to address issues such as uneven illumination and fish occlusion in real underwater environments, thereby achieving the identification of feeding and non-feeding states of fish in marine aquaculture environments. Monocular vision generally requires only a single camera, resulting in a relatively simple system configuration, lower implementation costs, and greater practicality for achieving satisfactory performance in real-world aquaculture scenarios.

However, monocular vision provides only 2D projection information and cannot directly acquire depth information. Therefore, when accurate 3D spatial information is required, such as fish spatial position, inter-individual distance, and the spatial relationship between fish and the feeding point, monocular vision may have certain limitations. To address these limitations, binocular vision-based methods for analysing fish feeding behaviour have gradually been developed. Binocular vision employs dual cameras to capture images and can reconstruct 3D features according to the principles of binocular vision, thereby overcoming the constraints of monocular vision. As a result, it allows for accurate and comprehensive acquisition of fish 3D features, offering significant advantages and considerable potential in the analysis of fish behaviour.

In recent years, there have been research achievements in fish behaviour analysis methods based on binocular vision. Somerton et al. [16] proposed the use of a benthic stereo camera system to observe and quantitatively analyse fish behaviour. This method employed benthic stereo cameras to obtain the precise 3D spatial coordinates of fish, and by analysing behavioural indicators such as swimming speed and direction, it ultimately completed the quantitative description of fish escape behaviour. Yu et al. [17] proposed a method that utilised splash phenomena generated during feeding to assess fish feeding intensity. In this method, binocular cameras were used to acquire splash images, and the splash area was calculated using a linear regression approach based on depth information. Subsequently, fish feeding intensity was assessed according to the variation trend of the splash area. More recently, the application of stereo vision in aquaculture has expanded from basic 3D localisation to 3D tracking and comprehensive behavioural monitoring. Li et al. [18] proposed a two-stage 3D multi-fish tracking model based on patch-based underwater stereo matching, enabling accurate 3D localisation and tracking of fish under complex underwater conditions. Zhang et al. [19] developed the FishKeypoints framework by integrating oriented fish detection, anatomical keypoint detection, and stereo vision, enabling the estimation of fish 3D positions, motion trajectories, body length, and distances to surrounding objects in commercial aquaculture environments.

However, existing methods generally analyse fish feeding intensity from the perspectives of texture features, behavioural characteristics, or even water splash patterns. In fact, when fish behaviour changes, their spatial distribution and structure also change accordingly. Therefore, analysing feeding intensity from the perspective of spatial features represents an interesting and reasonable approach. In our previous work, we utilised monocular vision technology to extract seven 2D spatial features of fish to assess their feeding intensity [20]. It achieved promising results compared with existing approaches, validating the rationality and effectiveness of assessing fish feeding intensity through spatial features. Nevertheless, we overlooked an important fact. Fish move in real 3D space, and their behaviour is not only characterised by vertical movements but also includes spatial motion features such as forward–backward and horizontal swimming. Two-dimensional images can only capture the projection of these movements onto the image plane and cannot obtain spatial position information or behavioural changes in the depth dimension, such as vertical movements and inter-individual distances. Depth information is crucial for accurately describing typical feeding behaviours, including aggregation and feed-chasing during the feeding process. However, monocular vision technology cannot provide such depth information and thus limits the accuracy of feeding intensity assessment to a certain extent. Considering these limitations, the binocular vision-based method is adopted in this study for fish feeding intensity assessment. Based on the principles of binocular vision, this technology allows the reconstruction of fish coordinates in 3D space, providing access to depth information and enabling an accurate description of spatial behavioural characteristics. However, when reconstructing 3D coordinates and obtaining depth information based on stereo vision principles, the reconstruction accuracy depends heavily on the matching of feature points between the binocular images. During feature point extraction, challenges such as inter-individual overlap and occlusion may lead to missed detections, resulting in missing feature points. These inconsistencies in the number and spatial positions of feature points between binocular images can adversely affect the acquisition of depth information. Consequently, fish feeding intensity assessment based on binocular vision remains highly challenging.

Based on the above analysis, we hypothesise that 2D and 3D spatial features provide complementary information, and that their fusion can improve the performance of fish feeding intensity assessment compared with using either 2D or 3D features alone. To test this hypothesis and address the aforementioned issues, a binocular vision-based assessment method for fish feeding intensity via 2D and 3D spatial feature fusion is proposed. First, binocular images of fish feeding are acquired from a lateral perspective. After segmenting fish targets and extracting feature points via image processing, the 2D spatial features are extracted. On this basis, the MPR method is proposed to restore and match missing feature points, and the WDT algorithm is employed to compute 3D spatial features. Finally, a TabPFN-based fish feeding intensity assessment model is constructed to achieve an accurate assessment of fish feeding intensity. The main contributions of this study are summarised as follows:

(1) A missing point restoration (MPR) method is proposed. By restoring the coordinates of missing feature points from the disparity maps of binocular images, it addresses the missing feature points caused by individual overlapping and occlusion, thereby enabling accurate matching of fish feature points.

(2) A weighted Delaunay triangulation (WDT) algorithm tailored for 3D space is introduced. By performing spatial triangulation to assign specific weights to feature points, it improves the accuracy of 3D spatial feature computation and effectively enhances the representational capacity of 3D spatial features for characterising fish feeding intensity.

(3) A novel fish feeding intensity assessment method is proposed, which integrates binocular vision with the fusion of 2D and 3D spatial features. By leveraging depth information, the proposed method improves the representation of fish spatial behaviour and supports fish feeding intensity assessment through the fusion of 2D and 3D spatial information.

## 2. Materials and Methods

### 2.1. Overall Process

**Figure 1:**
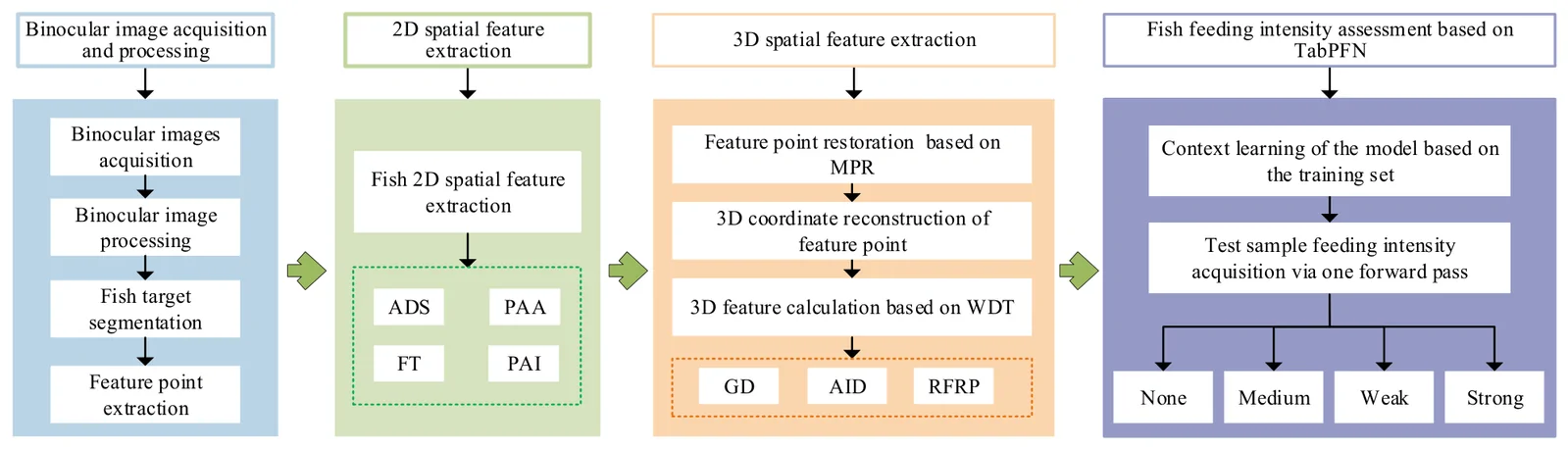
Overall process of binocular vision-based assessment method for fish feeding intensity via 2D and 3D spatial feature fusion.

A binocular vision-based assessment method via 2D and 3D spatial feature fusion is proposed in this study to achieve an accurate assessment of fish feeding intensity. It is illustrated in Figure 1.

**(1) Binocular image acquisition and processing.** A binocular camera was fixed to the side of the aquaculture tank to acquire binocular images of the fish. The images were then calibrated, rectified, and matched using standard stereo vision procedures. Image processing techniques were employed to segment the fish targets, and the centroid of each fish target was extracted as its feature point.

**(2) 2D spatial feature extraction.** Four 2D spatial features were extracted via image processing, namely the average distance from individuals to the water surface (ADS), fish tilt (FT), proportion of aggregation area (PAA), and proportion of aggregated individuals (PAI).

**(3) 3D spatial feature extraction.** Missing feature points in the binocular images were restored using the MPR algorithm, followed by feature point matching via the ORB algorithm. Based on the binocular vision principle, the 3D coordinates of the feature points were reconstructed. Finally, the WDT algorithm was introduced to compute 3D spatial features, namely group dispersity (GD), average inter-individual distance (AID), and relationship between fish and the feeding point (RFPR).

**(4) Fish feeding intensity assessment.** A TabPFN-based classification model is constructed to assess the fish feeding intensity accurately.

### 2.2. Binocular Image Acquisition and Processing

#### 2.2.1. Binocular Image Acquisition

**Figure 2:**
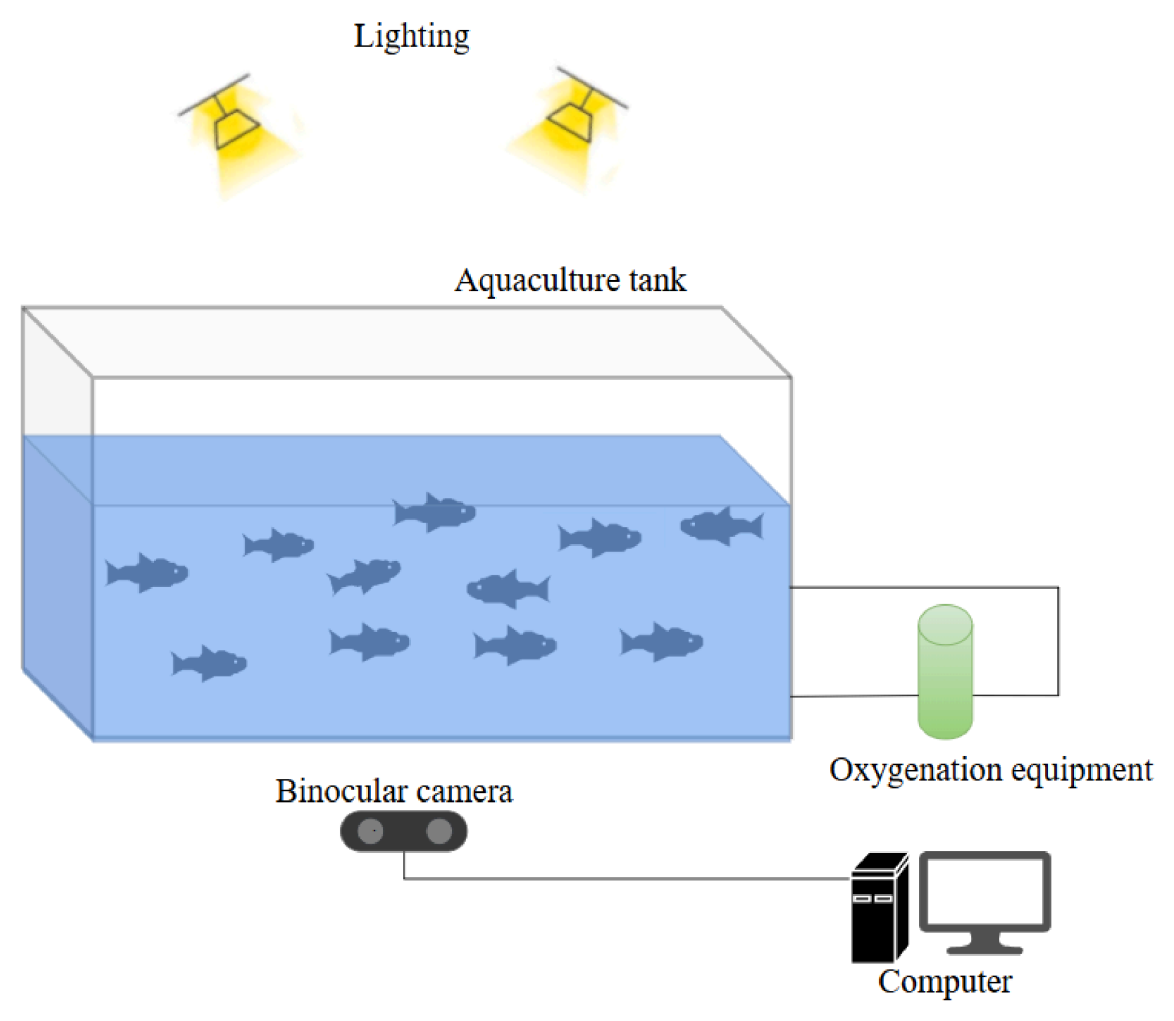
Experimental data acquisition platform.

The fish feeding intensity assessment experiment was conducted at Xuexing Building, Yangzhou University, Yangzhou, Jiangsu Province, China. The experimental data acquisition platform is shown in Figure 2, including a lighting system, an aquaculture tank (90 cm × 65 cm × 70 cm), oxygenation equipment (Sensen Group Co., Ltd., Zhoushan, China), a computer (Intel (R) Core (TM) i7-12650H CPU @ 2.30 GHz, 16.00 GB RAM, Hasee Computer Co., Ltd., Shenzhen, China), and a binocular camera (ZED 2i, Stereolabs SAS, San Francisco, CA, USA). A total of 15 juvenile Asian crucian carp (*Carassius auratus*) were initially maintained in the experimental tank. The same group of 15 fish was used throughout all video-recording sessions during the data-collection period. Their body lengths ranged from 15 to 25 cm, with an average body mass of approximately 80 g and a total wet biomass of approximately 1.20 kg. The water depth was 40 cm, corresponding to an initial stocking density of 64 fish m^−3^. The fish had been maintained in the experimental tank for more than one month before data collection and had fully acclimatised to the experimental environment. The experimental data were collected from 25 October to 13 December 2023. The experiment was conducted in a temperature-controlled indoor laboratory environment, where room-temperature regulation maintained the water temperature at approximately 20–24 °C throughout the experimental period. During the daytime, the laboratory was mainly exposed to natural daylight supplemented with artificial lighting, which was generally maintained until approximately 22:30, followed by the normal nighttime dark period. The dissolved oxygen concentration ranged from 5.8 to 7.5 mg L^−1^ and the pH ranged from 7.0 to 7.8 during the experiment. The binocular camera was mounted 50 cm from the side of the tank and 20 cm above the ground and was connected to a computer via a data cable for video recording.

A fixed-time, fixed-location, and fixed-quantity feeding strategy was adopted. Feeding was conducted twice daily at 9:00 am and 5:00 pm. During the routine husbandry period before formal data collection, the feeding amount was adjusted according to the actual feeding responses of the experimental fish, while considering the total fish biomass, husbandry conditions, and recommendations from experienced aquaculture personnel. After continued observation and adjustment, 60 pellets per feeding event were selected as the standardised feeding amount. This feeding amount was sufficient to elicit clear and complete feeding responses while minimising excessive uneaten feed that could interfere with subsequent behavioural observations. Using a fixed amount of 60 pellets also helped maintain consistent feeding conditions across independent feeding events. The pellets had a diameter of 3.0 mm, and 60 pellets had a total mass of approximately 3.0 g. The main ingredients of the pellets included fish meal, fish oil, soybean meal, peanut meal, rapeseed meal, wheat flour, vitamins, and minerals. With two feeding events per day, the total daily ration was approximately 6.0 g. Under the present experimental conditions, this feeding amount was sufficient to meet the daily feeding requirements of the fish and maintain normal husbandry conditions. To ensure comprehensive recording of feeding behaviour, video recordings lasted between 120 and 150 s on each occasion, covering the three stages of feeding, before, during and after. After excluding recordings affected by disturbances or incomplete feeding processes, 37 valid feeding videos were retained, corresponding to 37 independent feeding events.

**Figure 3:**
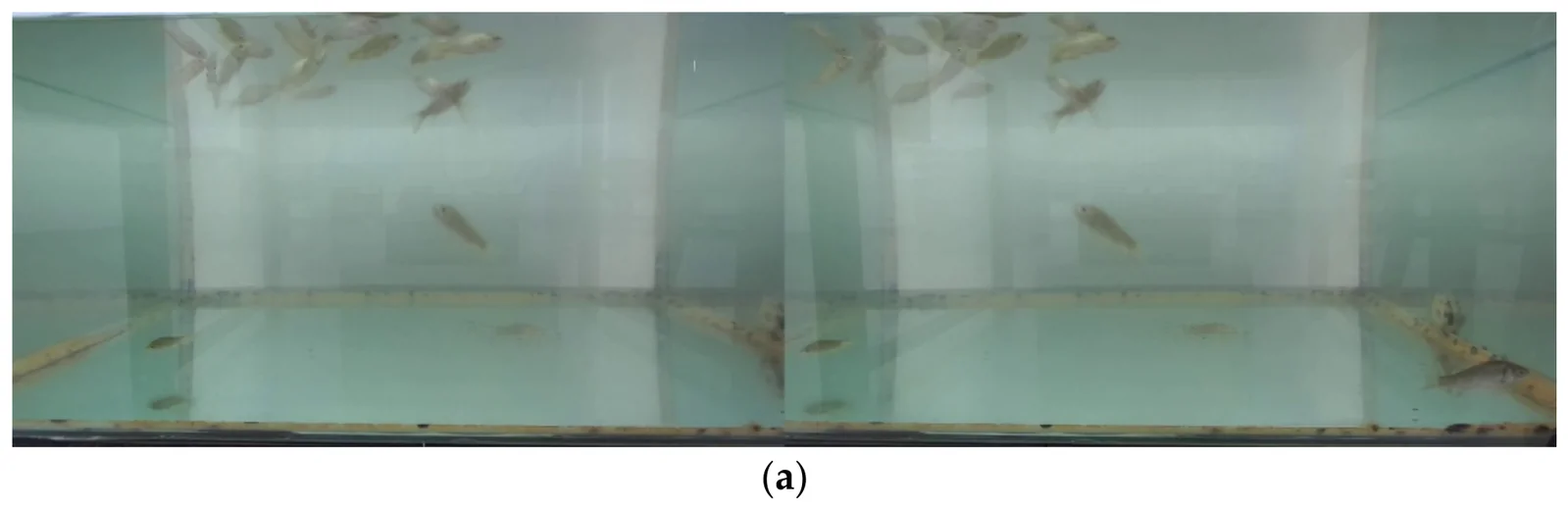

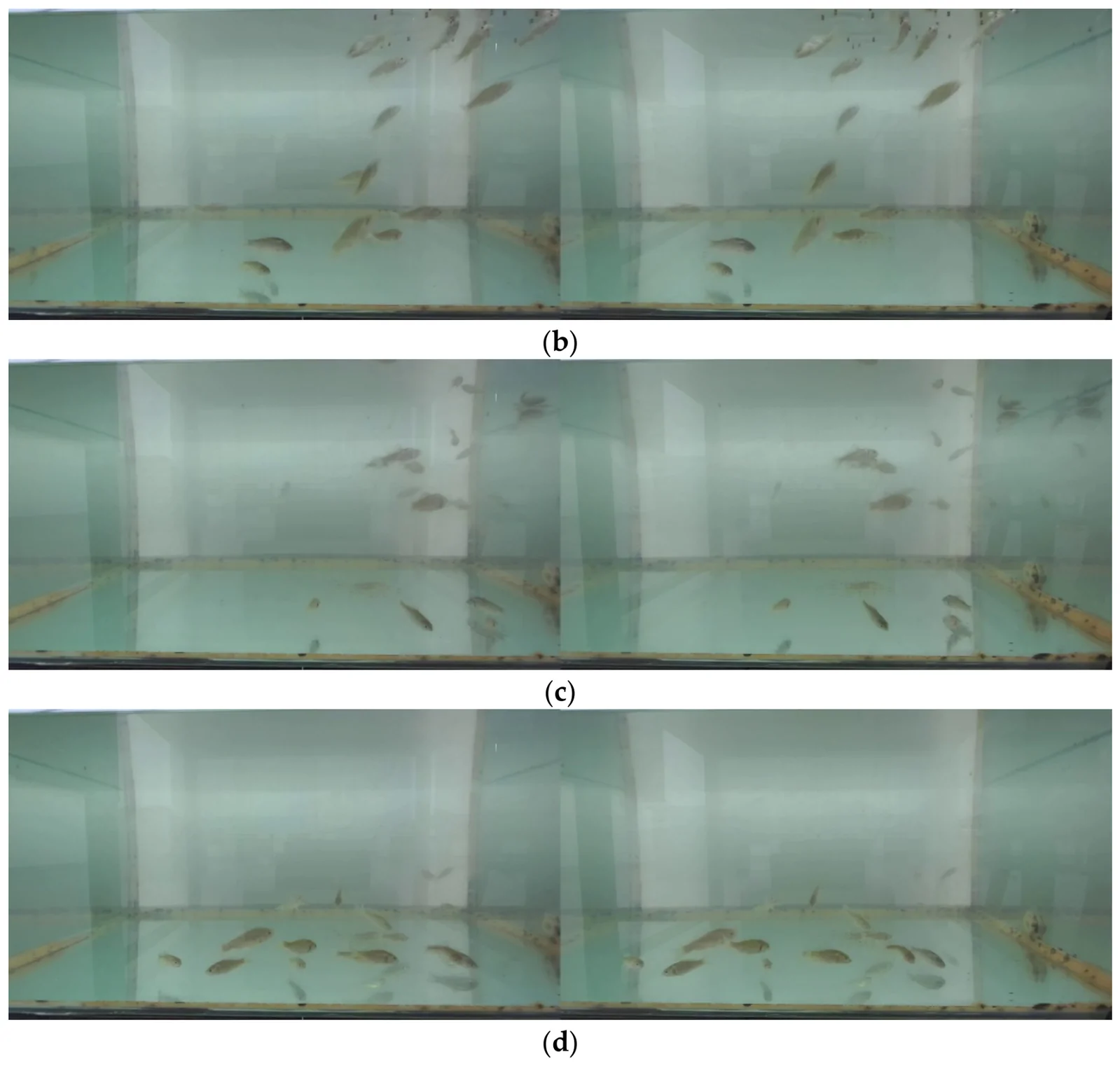
Binocular fish images. (**a**) none; (**b**) weak; (**c**) medium; (**d**) strong.

The video consists of 30 frames per second, with each frame representing a single image. Therefore, the dataset was constructed by extracting one frame from the video for every 15 frames. During data preprocessing, the extracted images were screened, and some highly similar consecutive frames were removed to reduce redundancy among adjacent images. After screening, a total of 9503 images with a resolution of 4416 × 1242 pixels were retained, as shown in Figure 3.

The collected fish feeding images were classified into four feeding intensity levels of none, weak, medium and strong [21]. The classification criteria were established with reference to previous studies [20] on fish feeding behaviour and were based on observable characteristics, including the fish response to feed, active feeding behaviour, and overall feeding state. To improve the reliability of the annotations, image labelling was performed by experienced aquaculture personnel in this study. Images for which the feeding state could not be clearly determined were excluded during data screening. The specific classification criteria are presented in Table 1. Subsequently, after completing feeding-intensity labelling, image preprocessing, and feature extraction, the resulting samples were split using a random seed of 42 into training/validation (75%, at a 4:1 ratio) and test (25%) sets. Table 2 provides detailed information on the partition.

#### 2.2.2. Fish Image Processing

To obtain clear fish targets and extract accurate spatial features, three preprocessing operations were applied to the acquired binocular images. Firstly, correction and matching were performed on the binocular images to eliminate image distortion and deformation. Second, image processing techniques were applied to segment clear fish targets. On this basis, the centroid of each fish target was extracted as a feature point to mark its position, thereby laying the foundation for extracting 2D and 3D spatial features of the fish.

**(1) Binocular image processing**

When capturing images using a binocular camera, the raw fish images may exhibit distortion and proportional imbalances due to lens distortion. We therefore need to process the binocular images through camera calibration, image correction and stereo matching to eliminate image distortion and extract the image disparity. The camera calibration and image rectification procedures followed the method described in our previous study [22]. Camera calibration was conducted using the binocular camera calibration module in MATLAB 2022 (The MathWorks, Inc., Natick, MA, USA). A 200 mm × 200 mm checkerboard calibration target with a 12 × 9 grid and a square size of 15 mm × 15 mm was used. A total of 25 stereo image pairs were acquired underwater at different orientations, with a resolution of 2208 × 1242 pixels for both the left and right images. Calibration images with a reprojection error greater than 0.5 pixel were excluded. Among the acquired calibration images, the maximum reprojection error was 0.66 pixel, and the mean reprojection error was 0.31 pixel. In addition, the stereo baseline was approximately 120 mm, and the intrinsic parameters (fx,fy,cx,cy) of the left and right cameras were (1067.04, 1067.08, 1111.34, 594.92) and (1065.51, 1065.47, 1125.05, 630.00) pixels, respectively. These parameters were then used to correct the images, restoring their original shape and proportions. Finally, the Semi-Global Block Matching (SGBM) algorithm [23] was employed to perform stereo matching on the binocular images. After binocular image rectification, SGBM first calculates the matching costs between the left and right images within the candidate disparity range. The matching costs are then aggregated along multiple directional paths, with smoothness constraints used to balance disparity continuity and depth discontinuities. Finally, the optimal disparity is selected according to the aggregated costs to generate the disparity map. The standard SGBM algorithm was adopted in this study without modification to its algorithmic structure. The main SGBM parameters were kept at their default settings, including numDisparities = 16, blockSize = 3, P1 = 0, P2 = 0, etc., with MODE_SGBM used as the matching mode.

**(2) Fish target segmentation**

Underwater fish images often suffer from uneven illumination and blurring, which may compromise the accuracy of feature extraction and ultimately affect the performance of feeding intensity assessment. To address these issues, background subtraction was first applied for image segmentation to isolate fish targets. Image enhancement was performed using linear transformation and histogram equalisation to improve the clarity of the target and reduce the impact of noise. Image binarization was subsequently performed to extract morphological and structural features of the fish targets. Finally, the findContours() function was used to detect and plot fish contours, providing reliable data input for subsequent feature point identification and spatial feature extraction. The main parameter settings were as follows: linear transformation was implemented using cv2.convertScaleAbs() with alpha = 1.2 and beta = 10; histogram equalisation was performed using cv2.equalizeHist(); image binarization was performed using Otsu’s thresholding method with thresh = 0, maxval = 255, and type = cv2.THRESH_BINARY + cv2.THRESH_OTSU; and contour detection was performed using cv2.findContours() with mode = cv2.RETR_EXTERNAL and method = cv2.CHAIN_APPROX_SIMPLE. These preprocessing procedures were implemented using standard image-processing functions without additional algorithmic modification or parameter optimisation.

Feature points refer to key points in an image that represent important information, such as shape and spatial position or exhibit significant variations. In this study, the centroid of each fish body was taken as the feature point to denote its spatial position. The centroid of the fish body can be obtained by computing the first-order moments of the connected component. The specific calculation procedure is as follows:

(1) $$m_{pq}=\sum_{j-1}^{N}\sum_{i-1}^{N}i^{p}k^{q}f(i,j)$$

where *m_pq_* is the (*p + q*)-th order moment of the image, *i* and *j* represent the pixel indices. The centroid coordinates of the fish target are given by (*x_c_, y_c_*).

(2) $$x_{c}=\frac{m_{10}}{m_{00}},y_{c}=\frac{m_{01}}{m_{00}}$$

Using the centroid of the fish as feature points enables the identification of the number and position of individual fish, thereby facilitating image feature extraction.

### 2.3. Two-Dimensional Spatial Feature Extraction

**Figure 4:**
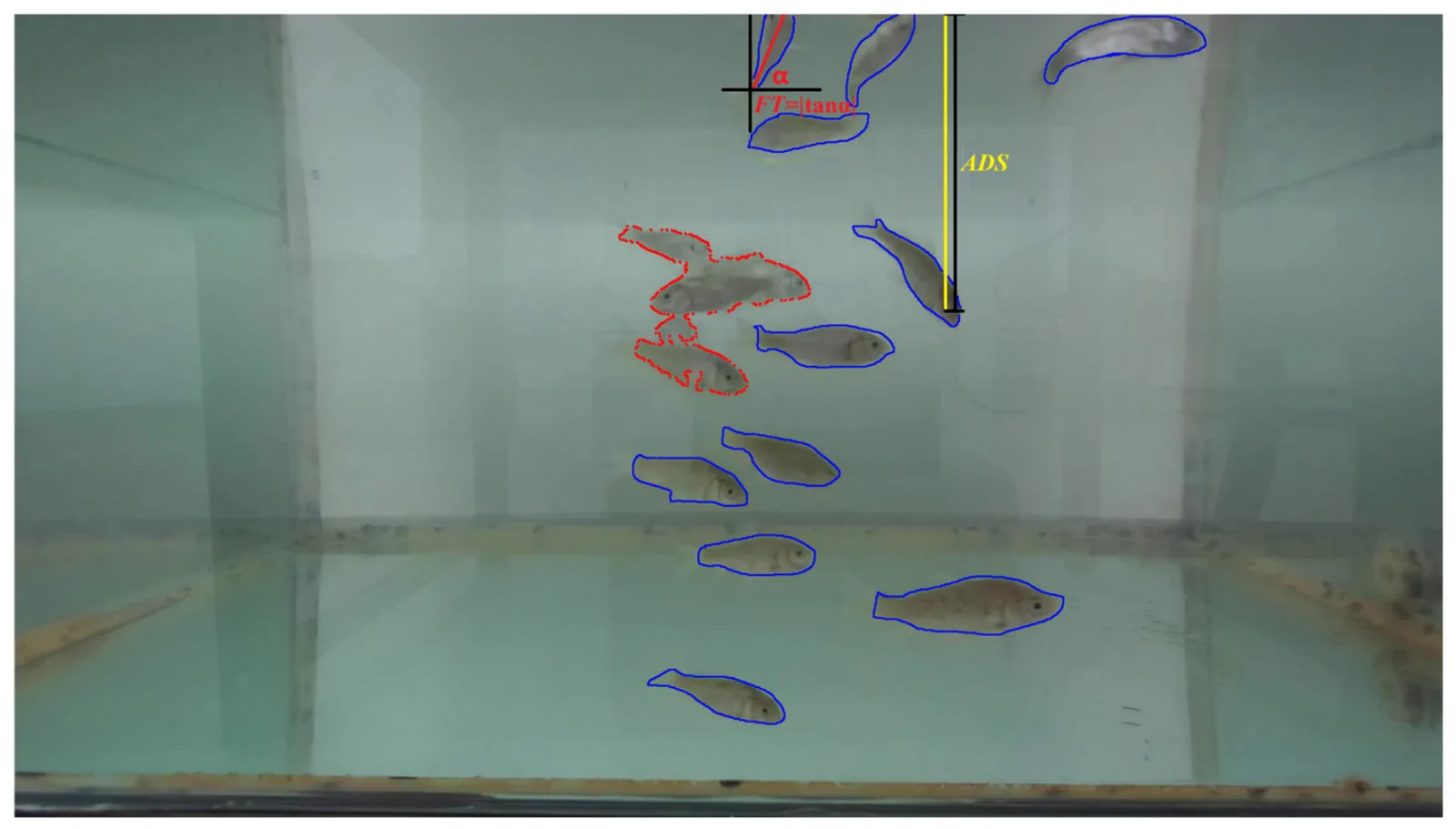
Schematic diagram of 2D spatial features.

Feature extraction refers to the process of extracting representative information from images that can describe their content, structure or attributes. When fish feeding intensity changes, their position, distribution, aggregation, and posture also change accordingly. Therefore, four spatial features proposed in our previous work [20] are extracted to assess fish feeding intensity, namely the average distance from individuals to the water surface (ADS), fish tilt (FT), proportion of aggregation area (PAA), and proportion of aggregated individuals (PAI). The schematic illustration of these features is shown in Figure 4.

The distance from an individual to the water surface is defined as the distance from the feature point to the water surface, as illustrated by the yellow line in Figure 4, and this distance corresponds to the length of the yellow line (unit: px), denoted as ADS. In aquaculture, fish in the none state typically swim in the middle and lower layers of the water, whereas the broadcast feeding method forces fish to surface for food. Generally, higher feeding intensity increases both the number of surfacing individuals and the degree of upward movement, resulting in a smaller ADS. Therefore, the feature ADS can, to some extent, characterise fish feeding intensity. The ADS is calculated as follows:

(3) $$ADS=\frac{\sum_{1\leq i\leq N}y_{i}}{N}$$

where *i* denotes the *i*-th individual, *y_i_* represents the vertical distance from the *i*-th fish feature point to the water surface, and *N* denotes the total number of individuals in the image, that is the total number of feature points.

The fish tilt is defined as the absolute value of the tangent of the angle between the line connecting the head and tail of the fish and the horizontal line, as illustrated by the red line in Figure 4, and FT denotes the average fish tilt of all individuals. Fish typically swim in a horizontal posture under non-feeding conditions, whereas during feeding at the water surface, they exhibit a tilted posture with the head above the surface and the body submerged [24,25]. Therefore, the feature FT tends to increase during feeding. It is calculated as follows:

(4) $$FT=\frac{\sum_{1\leq i\leq N}\left|\frac{y_{i2}-y_{i1}}{x_{i2}-x_{i1}}\right|}{N}$$

where (*x_i_*_1_*, y_i_*_1_) denote the coordinates of the fish head, (*x_i_*_2_*, y_i_*_2_) denote the coordinates of the fish tail, and *N* denotes the total number of individuals in the image.

Proportion of aggregation area (PAA) is the ratio of the aggregation area to the total area of fish connected components, while proportion of aggregated individuals (PAI) is the ratio of the number of individuals in the aggregation area to the total number of individuals in the image. After binarization, a binary image is obtained, the fish connected components in the image are classified into two categories of aggregation and non-aggregation. If a connected component contains two or more individuals, it is classified as an aggregation area (the red contour region in Figure 4). Otherwise, it is regarded as a non-aggregation area (the blue contour region in Figure 4). Under non-feeding conditions, fish swim freely and are relatively dispersed, whereas under feeding conditions, fish tend to aggregate around the feed and compete for food. Generally, the stronger the feeding intensity, the more pronounced the aggregation and competition, leading to larger proportions of aggregation area and aggregated individuals relative to their respective totals. Based on this, the features PAA and PAI are extracted to characterise the aggregation behaviour of fish. The calculation methods are given as follows.

(5) $$PAA=\frac{A_{1}}{A},PAI=\frac{N_{1}}{N}$$

where *A*_1_ denotes the area of the aggregation region, *A* denotes the total area of fish connected components, *N*_1_ denotes the number of individuals within the aggregation region, and *N* denotes the total number of individuals in the image.

In Figure 4, there is one aggregation area and eleven non-aggregation areas, the aggregation area contains five individuals, while the number of non-aggregated individuals is eleven, therefore *PAI =* 5/(11 + 5) = 0.3125.

### 2.4. Three-Dimensional Spatial Feature Extraction

Although 2D spatial features can provide certain behavioural information of fish, they are limited to planar representations and lack depth information in 3D space, thereby preventing accurate characterisation of the true spatial behaviour and state of fish. Therefore, for more accurate fish information and spatial feature analysis, three 3D spatial features, namely group dispersity (GD), average inter-individual distance (AID), and relationship between fish and the feeding point (RFPR), are extracted for fish feeding behaviour analysis.

**Figure 5:**
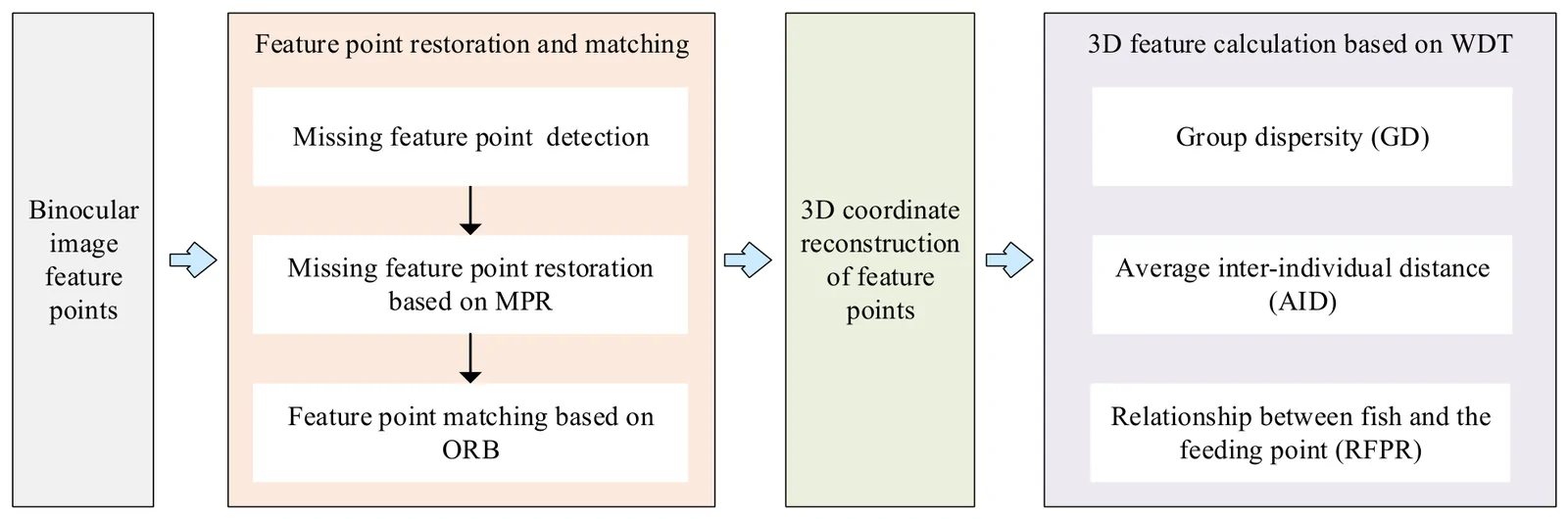
Flowchart of 3D spatial feature extraction.

The process for extracting 3D spatial features is illustrated in Figure 5. First, the feature point information from binocular images is read, and feature point matching based on the MPR method is performed, thereby restoring missing feature points and establishing corresponding matches. Subsequently, the 3D coordinates of feature points are reconstructed based on the matching results. On this basis, the WDT algorithm is employed to accurately obtain 3D fish features, such as group dispersity and average inter-individual distance. This method addresses the problem of matching failure caused by missing feature points, and enhances the representation capability of fish spatial dispersity and by introducing the WDT algorithm, which is highly beneficial for accurate assessment of fish feeding intensity.

#### 2.4.1. Feature Point Restoration Based on MPR

**Figure 6:**
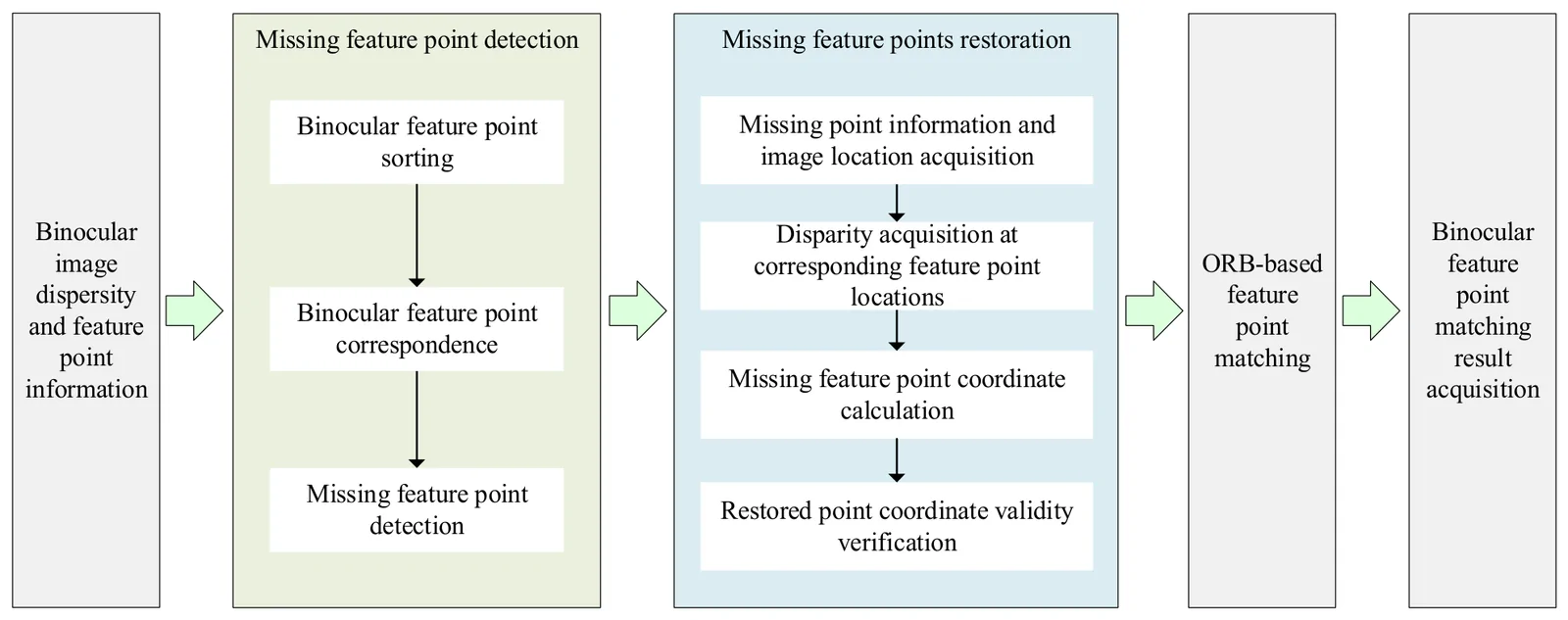
Flowchart of feature point restoration and matching.

Feature point matching refers to the process of identifying corresponding feature points between two images and establishing their correspondences. Accurate feature point matching is essential for reconstructing 3D coordinates and is crucial for extracting 3D spatial features of fish. However, fish feeding images acquired by binocular cameras are often affected by inter-individual overlap and occlusion, which may lead to missed detections and consequently result in missing feature points. Traditional feature point matching algorithms are susceptible to such missing points, which may cause matching failures and prevent accurate reconstruction of spatial information, affecting the accuracy of 3D feature extraction. To address this issue, a feature point restoration method based on MPR is proposed in this study. It can estimate the coordinates of missing feature points based on binocular disparity, which is important to restore the missing feature points and achieve accurate matching of feature points between binocular images. It should be noted that the missing-point coordinates reconstructed by MPR are algorithmically estimated from the spatial positions and disparity information available in the binocular images, rather than being manually assigned according to the feeding-intensity category. The restoration process does not use feeding-intensity labels, classification results, or other class-related information, and the same restoration procedure is applied to samples from all feeding-intensity levels. The specific process is shown in Figure 6.

First, the feature points in the binocular images and their corresponding disparity values are obtained, and the feature points in the left and right images are sorted according to their spatial order. Taking the feature points in the left image as reference, the corresponding disparity values are used to predict the coordinates of matching points in the right image, and a search is performed within a neighbourhood centred on the predicted point to locate actual feature points. If a match is found, an initial matching pair is established; otherwise, the corresponding position in the right image is marked as a missing feature point. Similarly, taking the feature points in the right image as reference, missing feature points in the left image are detected in a reverse manner. On this basis, for the detected missing feature points, their spatial coordinates are inversely computed using the disparity consistency principle based on the matched points and disparity values in the corresponding image, thereby restoring the missing feature points. The validity of the restored points is then verified by evaluating the spatial distance and distribution consistency between the restored points and neighbouring normal feature points. Finally, the original feature points and the validated restored feature points are jointly input into the ORB feature point matching algorithm [26] to complete the matching of feature points between binocular images. The pseudocode is presented in Algorithm 1:
**Algorithm 1** Missing point restoration**Input**: Left feature point set L, Right feature point set R**Output**: Missing point set M1: **for** each point *p_i_* ∈ L, R **do**2:      find the point corresponding to *p_i_* and record information Info;3:            if there is no point corresponding to *p_i_* 4:                  N[*i*] = *p_i_*; 5: **end for**6: **for** each point *n_i_* ∈ N **do** 7:      confirm again that there is no point corresponding to *n_i_*; 8:      restore the point *m_i_* corresponding to *n_i_* based on Info; 9:            M[*i*] = *m_i_*10: **end for** 11: Return M

#### 2.4.2. Three-Dimensional Coordinate Reconstruction of Feature Points

**Figure 7:**
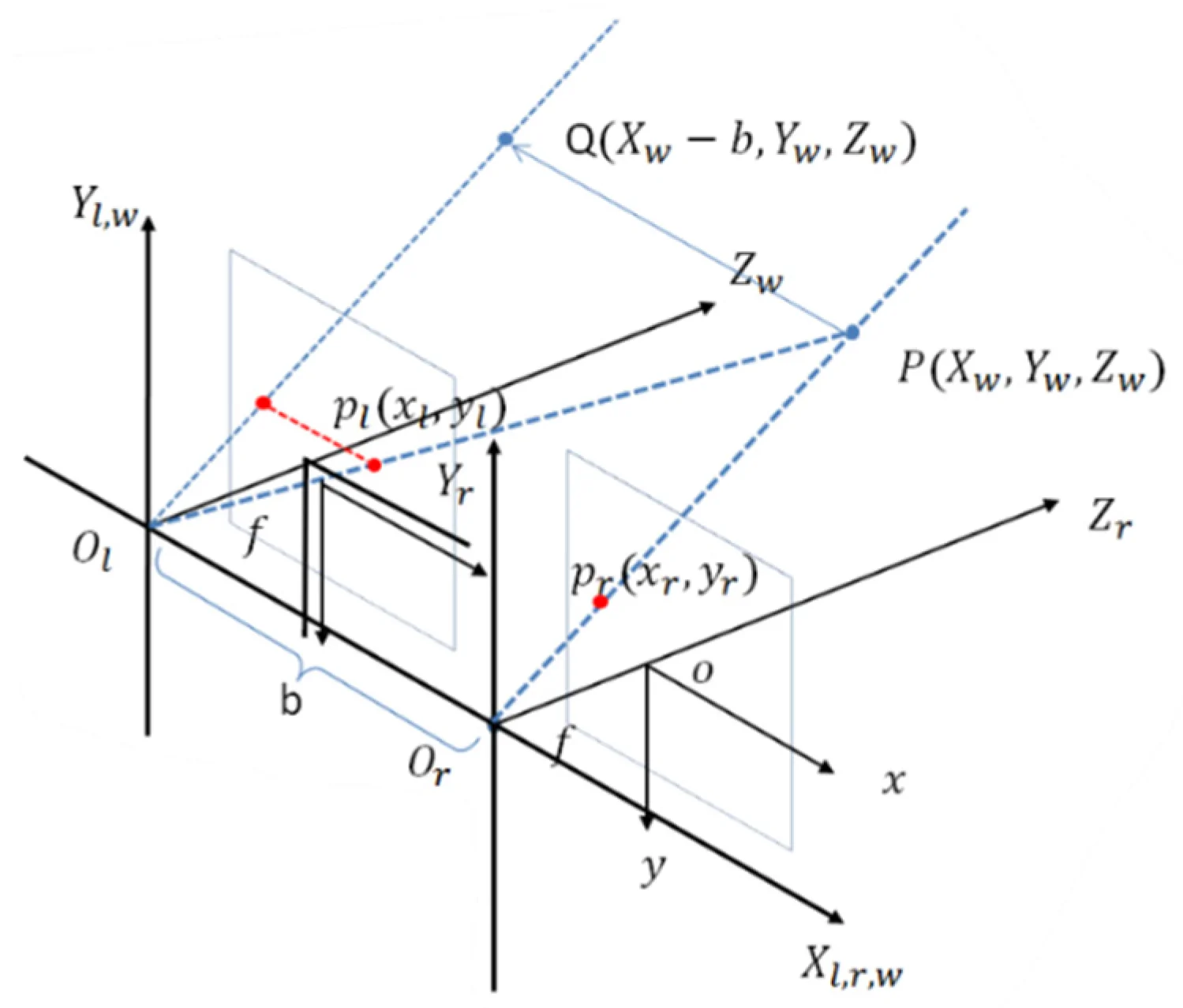
Principle of binocular stereoscopic vision. The blue dotted lines indicate the projection lines used for binocular triangulation, while the red dotted line is an auxiliary line illustrating the geometric relationship between the projected points.

Based on the results of feature point matching, the 3D coordinates of feature points are reconstructed through the principles of binocular vision, which serves as the foundation for extracting 3D spatial features of fish. The principle of binocular vision is shown in Figure 7.

In Figure 7, *f* denotes the focal length of the camera, *b* denotes the baseline length, and *d* denotes the disparity between the corresponding points in the left and right images. Accordingly, the following relationships can be obtained:

(6) $$x_{l}=f\times \frac{X_{w}}{Z_{w}},x_{r}=f\times \frac{X_{w}-b}{Z_{w}},y_{l}=y_{r}=y=f\times \frac{Y_{w}}{Z_{w}}$$

Therefore, the 3D coordinates of point *P* (*X_w_*, *Y_w_*, *Z_w_*) in space can be computed from its corresponding coordinates *p_l_* and *p_r_* in the left and right images as follows:

(7) $$X_{w}=b\times \frac{x_{l}}{d},Y_{w}=b\times \frac{y}{d},Z_{w}=f\times \frac{b}{d}$$

where *b* denotes the baseline length of the binocular camera, *x_l_* represents the horizontal coordinate of point *P* in the left image, *d* denotes the disparity of the binocular images, *y* represents the vertical coordinate of point P in the images, and *f* denotes the focal length of the binocular camera.

#### 2.4.3. Three-Dimensional Feature Calculation Based on WDT

After reconstructing the 3D spatial coordinates of fish feature points, the spatial positions of fish in the 3D aquatic environment are obtained. Accordingly, the 3D coordinate of the *i*-th fish, *p_i_* can be expressed as:

(8) $$p_{i}=\left(x_{i},y_{i},z_{i}\right)$$

Thus, the distance between two feature points can be expressed as:

(9) $$d_{ij}=\sqrt{{\left(x_{i}-x_{j}\right)}^{2}+{\left(y_{i}-y_{j}\right)}^{2}+{\left(z_{i}-z_{j}\right)}^{2}}$$

where *d_ij_* denotes the 3D distance between the *i*-th and *j-th* individuals, *x, y* and *z* represent the horizontal, vertical and height coordinates of a point in the 3D space, respectively.

In aquaculture, feed is typically provided at fixed locations; therefore, fish tend to aggregate around the feeding point during feeding, whereas under none feeding conditions, fish swim freely and are more dispersed. Consequently, the occurrence of aggregation during feeding implies that the average inter-individual distance decreases significantly compared with the non-feeding state, and group dispersity also decreases accordingly. Furthermore, during feeding, pellets were observed to move downward in the water column, and fish generally exhibit a behavioural pattern of first feeding below the feeding point and then gradually rising towards the water surface. According to the above 3D spatial information and behavioural characteristics, three features of GD, AID, and RFPR are extracted in this study to assess fish feeding intensity.

**(1) Group dispersity (GD)**

Group dispersity (unit: mm^2^) refers to the degree of dispersion within a fish school. Generally, a larger dispersity indicates a higher level of dispersion, whereas a smaller value indicates a more aggregated distribution. To accurately characterise group dispersity, a WDT algorithm suitable for 3D space is proposed. In this approach, feature points in space are treated as vertices of Delaunay tetrahedra for spatial triangulation, and specific weights are assigned based on the participation of triangles in the triangulation network, thereby enabling the obtained data to more accurately describe group dispersity. The calculation of the weights is given as follows.

**Figure 8:**
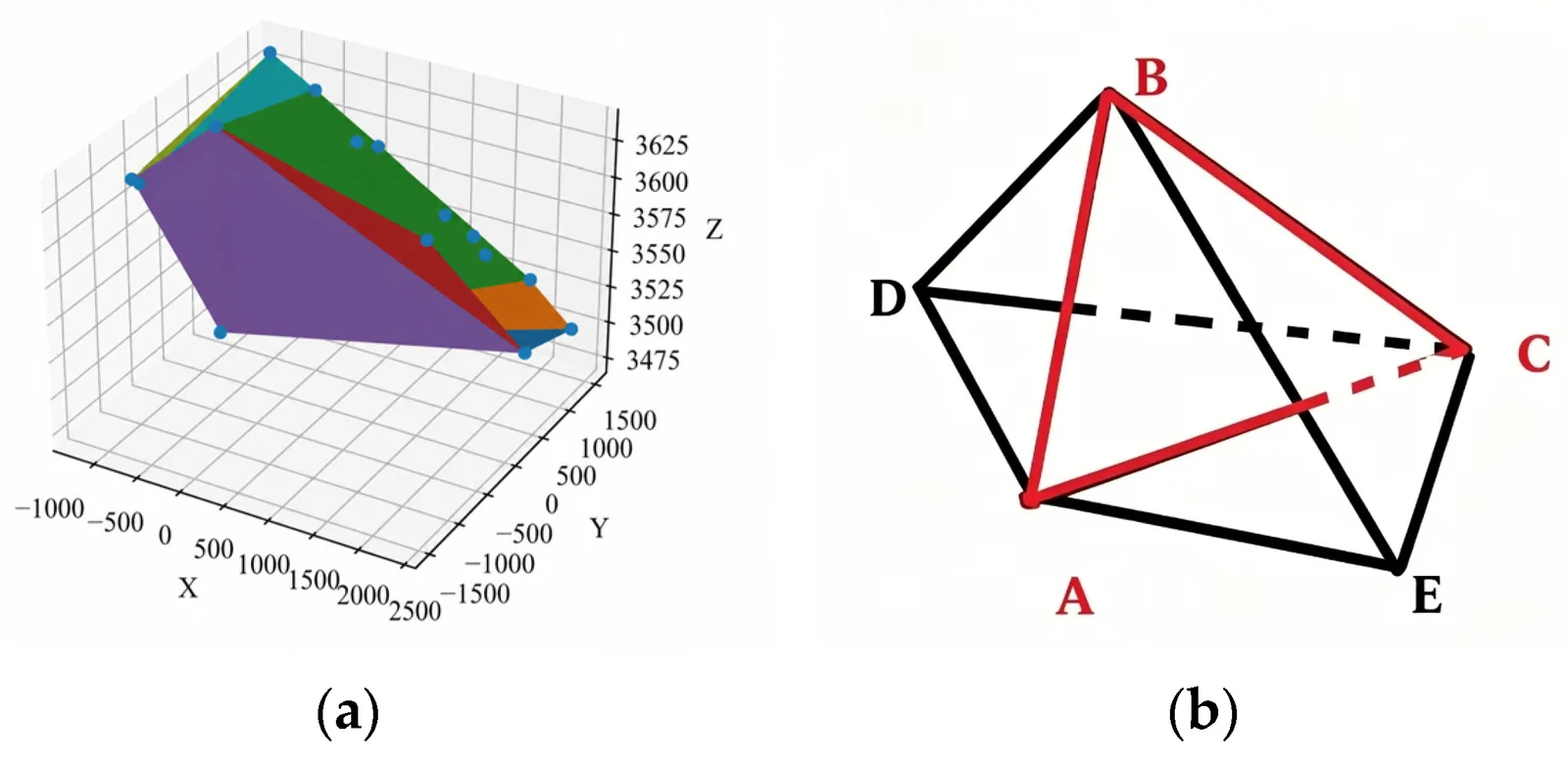
Weighted Delaunay triangulation diagram. (**a**) Example of fish WDT result; (**b**) WDT basic principle. The colors in (**a**) are used only to distinguish different triangular faces. In (**b**), A–E denote feature points, and triangle ABC represents the shared triangular face, while the remaining triangular faces are boundary faces.

After performing triangulation on points in 3D space, multiple tetrahedral structures are formed, as shown in Figure 8. In the tetrahedral mesh, for a point *A*, there are five triangular faces containing point *A*, namely triangles ABC, ACD, ACE, ABD and ABE. Triangles such as ABC, which form part of two tetrahedra, are defined as shared triangles, and their number is denoted as *b*. The remaining triangles are regarded as boundary triangles, and their number is denoted as *a*. For each feature point, the numbers of associated boundary and shared triangular faces are counted in the same manner, and the corresponding coefficient *k* is calculated as:

(10) $$k=1+\frac{a-b}{a+b}$$

where the constant term 1 represents the initial weight of all feature points, and $\frac{a-b}{a+b}$ characterises the relative difference between the numbers of boundary and shared triangular faces in the local structure. The relative numbers of these two types of triangular faces are used to describe differences in the local mesh structure. When *a* = *b*, *k* = 1, representing the reference case. When *a* > *b*, it indicates that the number of boundary triangles is larger, meaning that the feature point is located at the boundary of the fish school. Accordingly, the weight *k* increases, thereby enhancing the contribution of boundary individuals and improving the sensitivity to variations in boundary features. Conversely, when *a* < *b*, it indicates that the number of shared triangles is larger, meaning that the feature point is located within the aggregation region. In this case, the weight *k* decreases accordingly to mitigate abnormal feature values caused by repeated counting of multiple feature points. Thus, the coefficient *k* is determined according to the relative numbers of boundary and shared triangular faces around the feature point. This weighting strategy is designed to reflect the local topological position of each feature point within the tetrahedral mesh, with greater emphasis placed on boundary-related spatial variation.

After obtaining the corresponding coefficient *k_i_* for each feature point, *k_i_* is applied to the corresponding surface area *A_i_*, and the resulting values are averaged over all *N* feature points to obtain GD. Through this approach, the sensitivity of the tetrahedral mesh to local feature variations is enhanced, thereby enabling a more accurate representation of the spatial dispersity of the fish school. The feature GD is calculated as follows:

(11) $$GD=\frac{\sum_{1\leq i\leq N}\left(k_{i}\times A_{i}\right)}{N}$$

where *N* denotes the total number of individuals in the image, *k_i_* represents the weight coefficient of the *i*-th point, and *A_i_* denotes the surface area of the tetrahedra in which the *i*-th point participates.

**(2) Average inter-individual distance (AID)**

The average inter-individual distance (unit: mm) refers to the mean distance between all pairs of individuals in the fish school. Based on the aggregation behaviour observed during feeding, this feature is used to assess fish feeding intensity. It is calculated as follows:

(12) $$AID=\frac{2}{N\times (N-1)}\times \sum_{1\leq i<j\leq N}d_{ij}$$

where *d_ij_* denotes the distance between the *i*-th and *j*-th individuals, and *N* denotes the total number of individuals in the image.

**(3) Relationship between fish and the feeding point (RFPR)**

The relationship between fish and the feeding point is defined by the distance to the point and the inclination angle of the connecting line. During the feeding process, pellets were observed to move downward from the feeding point, and fish typically feed below the point before rising to the surface [27]. Based on this behaviour, the RFPR feature is used to characterise the distance–inclination relationship between the fish and the feeding point. For each individual, the corresponding relationship value is calculated based on its distance to the feeding point and the inclination angle of the connecting line, and RFPR is then obtained by averaging the calculated values over all individuals in the image, as follows:

(13) $$RFPR=\frac{\sum_{1\leq i\leq N}\left(dist_{i}\times \sqrt{r_{i}\times 2}\right)}{N}$$

where *dist_i_* denotes the distance between the *i*-th individual and the feeding point, *r_i_* represents the inclination angle of the line connecting the individual and the feeding point, and *N* denotes the total number of individuals in the image.

### 2.5. Fish Feeding Intensity Assessment Based on TabPFN

**Figure 9:**
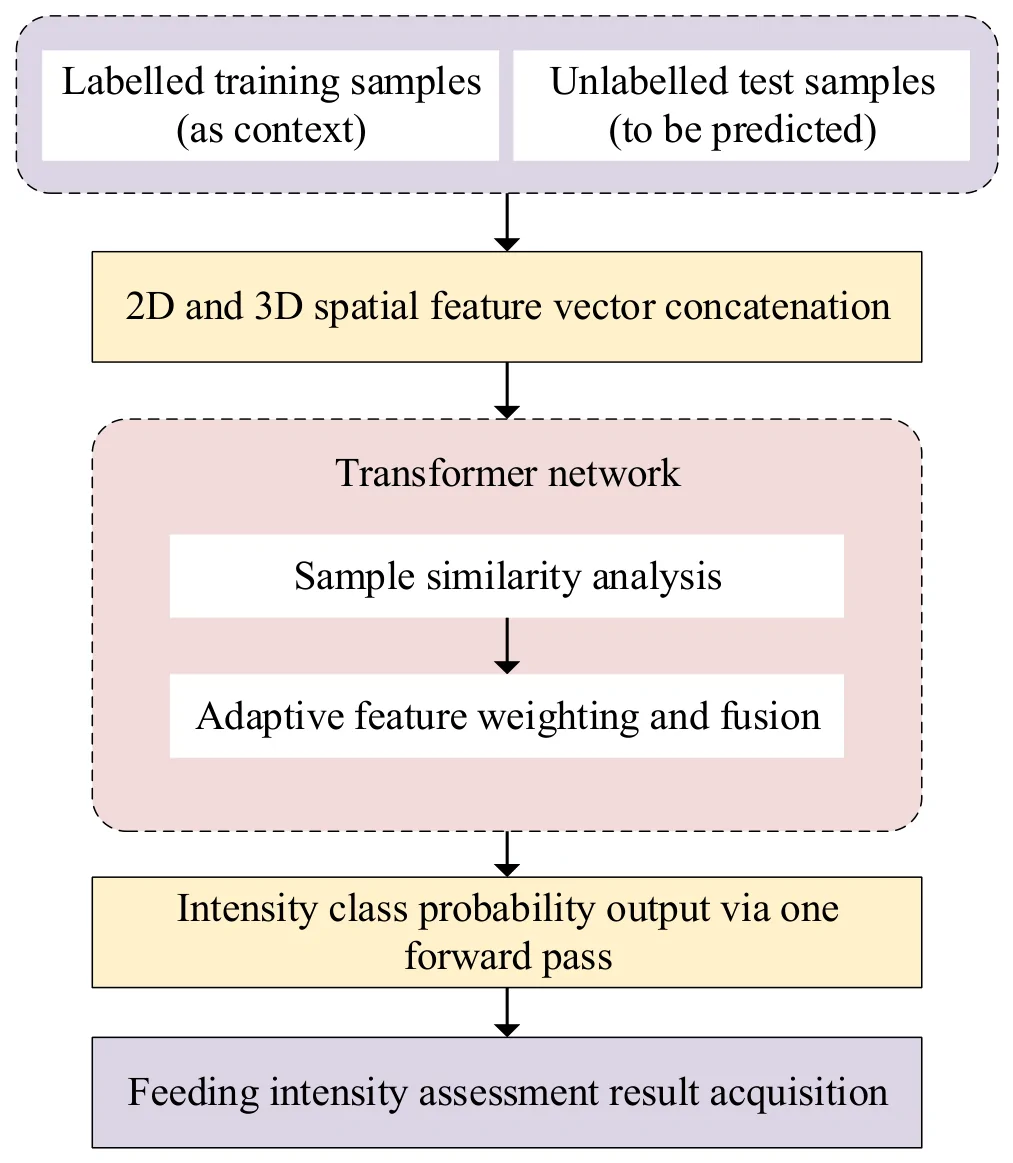
Flowchart for fish feeding intensity assessment based on TabPFN.

Based on the extracted 2D and 3D fish features, a classification model is built to achieve fish feeding intensity assessment. TabPFN is a “one-shot learning” foundation model specifically designed for tabular data [28]. It is a neural network based on the Transformer architecture. After a one-time pre-training on a large-scale synthetic dataset, all parameters are frozen. In this study, TabPFN v6.4.0 was used for fish feeding intensity assessment. During inference, the labelled training samples are provided as context, and the model directly predicts the labels of test samples in a single forward pass without any parameter updates. Therefore, no gradient-based parameter optimisation or additional training was performed for TabPFN in the present study, and early stopping was not applicable. This model offers high speed and robustness, providing computational efficiency for fish feeding intensity assessment. Therefore, it is selected as the fish feeding intensity assessment model in this study. The workflow of using TabPFN for fish feeding intensity assessment is illustrated in Figure 9.

The four extracted 2D and three 3D spatial features are concatenated to form a 7-dimensional fused spatial feature vector. During spatial-feature extraction and calculation, a small number of samples contained missing values because some spatial features could not be assigned valid numerical values. Samples with substantial missing information that prevented the acquisition of valid spatial features were removed during data screening. Samples containing only a small number of missing feature values were retained, and no additional manual imputation was performed; instead, the built-in missing-value handling mechanism of TabPFN was used. The training set, comprising the 7-dimensional features and their corresponding feeding intensity labels, is then provided as context to the TabPFN model, together with the unlabelled test samples. During this process, the multi-head attention mechanism of the Transformer-based TabPFN automatically analyses the similarity relationships between the test samples and the training samples, and adaptively assigns weights to the 2D and 3D features to achieve deep feature fusion. Ultimately, TabPFN outputs the probability distribution of the test samples belonging to the four feeding intensity classes through a single forward pass, enabling rapid and accurate feeding intensity assessment and providing a basis for subsequent precision feeding decisions.

### 2.6. Evaluation Metrics

The following five metrics are used to evaluate the performance of the proposed method.

(14) $$Accuracy=\frac{TP+TN}{TP+FP+TN+FN}$$

(15) $$Precision=\frac{TP}{TP+FP}$$

(16) $$Recall=\frac{TP}{TP+FN}$$

(17) $$Specificity=\frac{TN}{FP+TN}$$

(18) $$F1-score=2\times \frac{Precision\times Recall}{Precision+Recall}$$

where TP, TN, FP, and FN represent true positives, true negatives, false positives, and false negatives, respectively. It should be noted that, for the four-class feeding-intensity classification task in this study, specificity was calculated using a one-vs-rest strategy. For each class, the current class was treated as the positive class, while the remaining three classes were collectively treated as the negative class, and specificity was calculated as $\frac{TN}{FP+TN}$. For the four-class task, precision, recall, specificity, and F1-score were calculated separately for each feeding-intensity class, and the reported average values were obtained as the unweighted arithmetic mean of the four class-specific values. Accuracy was calculated globally as the proportion of correctly classified samples among all test samples.

## 3. Results

### 3.1. Experiment Setup

The experimental hardware environment comprised the Windows 11 operating system, an Intel^®^ Core™ i7-12650H CPU (16 cores, 2.3 GHz), 32 GB of DDR5 RAM, and an NVIDIA GeForce RTX 4060 Laptop GPU (8 GB VRAM). The software platform consisted of the Python 3.9, PyTorch 2.6.0 with CUDA 12.4, OpenCV 4.7, and scikit-learn 1.6.1.

To ensure a fair comparison, the proposed model and the comparison models shared the same input features, dataset partition, and evaluation metrics. For TabPFN, the extracted seven-dimensional spatial features were directly used as model inputs without additional manual missing-value imputation or feature scaling. For comparison models requiring iterative training, the validation set was used for training-process monitoring, model selection, and early stopping where applicable, whereas the test set was used only for final performance evaluation. For comparison models employing early stopping, the patience was set to 10 epochs. Training was terminated if the monitored validation performance showed no improvement for 10 consecutive epochs, and the best-performing model weights were retained. In addition, training was stopped when the variation in the monitored metric remained below 0.15% for 10 consecutive epochs. The comparison models included TabNet, eXtreme Gradient Boosting (XGBoost), Light Gradient Boosting Machine (LGBM), Random Forest (RF), Multi-Layer Perceptron (MLP), Decision Tree (DT), and 1D Convolutional Neural Network (1D-CNN). The main hyperparameter settings of XGBoost, LGBM, RF, MLP, DT, and 1D-CNN followed those used in our previous study [20]. For clarity, the detailed parameter settings are provided in Table 3. Parameters not specified in Table 3 were retained at the default settings of the corresponding software packages.

### 3.2. Image Processing Results

**Figure 10:**
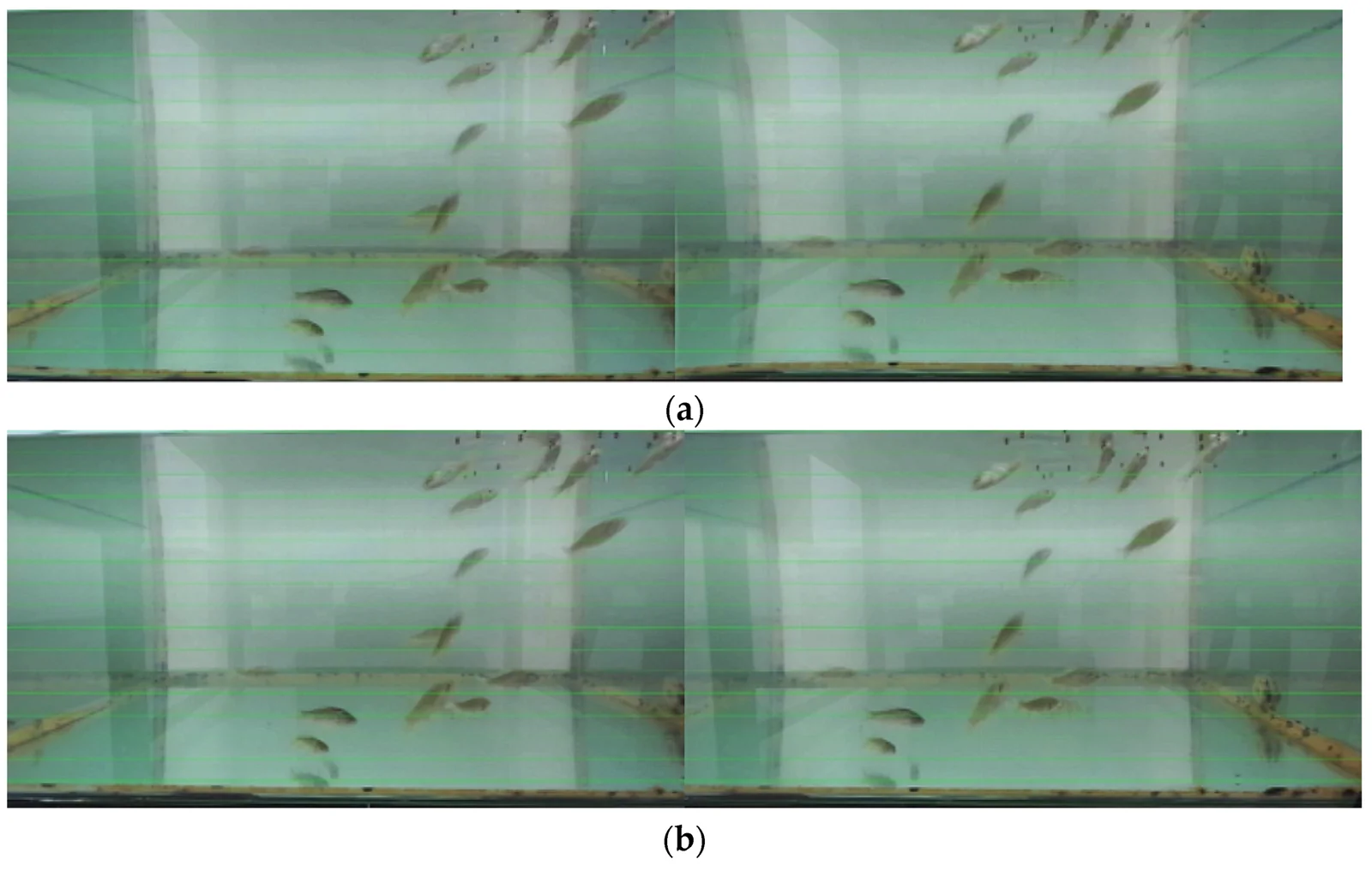
Comparison before and after correction. (**a**) Before correction; (**b**) After correction.

Image correction and stereo matching were performed on the binocular fish images to eliminate image distortion. A comparison before and after correction is shown in Figure 10.

From Figure 10a, it can be observed that the images before correction exhibit significant distortion, and the imaging planes of the left and right cameras are not aligned on a common horizontal line, which may affect subsequent image processing and feature point matching. The corrected images, as shown in Figure 10b, successfully eliminate distortion, aligning the imaging planes of the two cameras on the same horizontal line.

**Figure 11:**
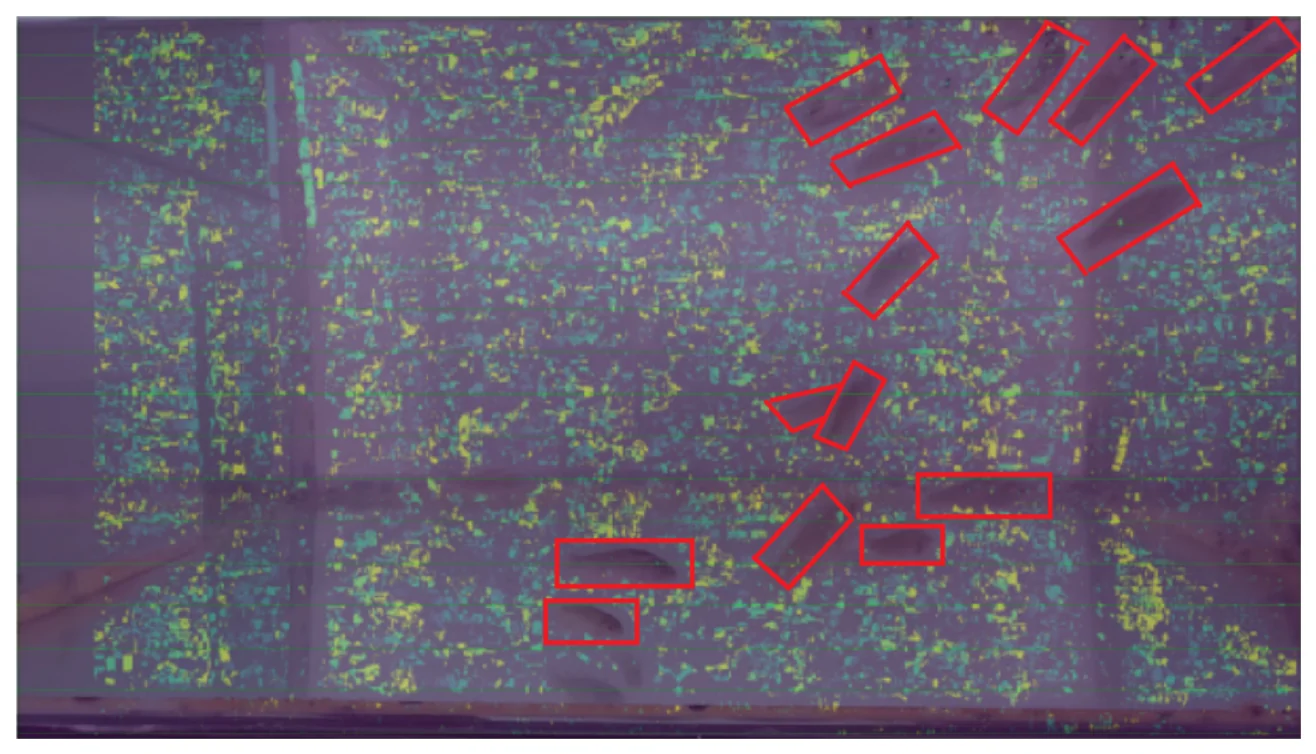
Disparity map obtained by SGBM stereo matching.

After the fish images were corrected, stereo matching was performed on the images to obtain the disparity map, as shown in Figure 11.

A red rectangular box is used to highlight the regions corresponding to individual fish in the disparity map. From Figure 11, it can be seen that the regions corresponding to fish appear darker and contain fewer noise points compared with other regions, enabling a clear distinction between fish and the image background. At this stage, the disparity values of the binocular images are obtained, which provide sufficient disparity information for feature point extraction.

**Figure 12:**
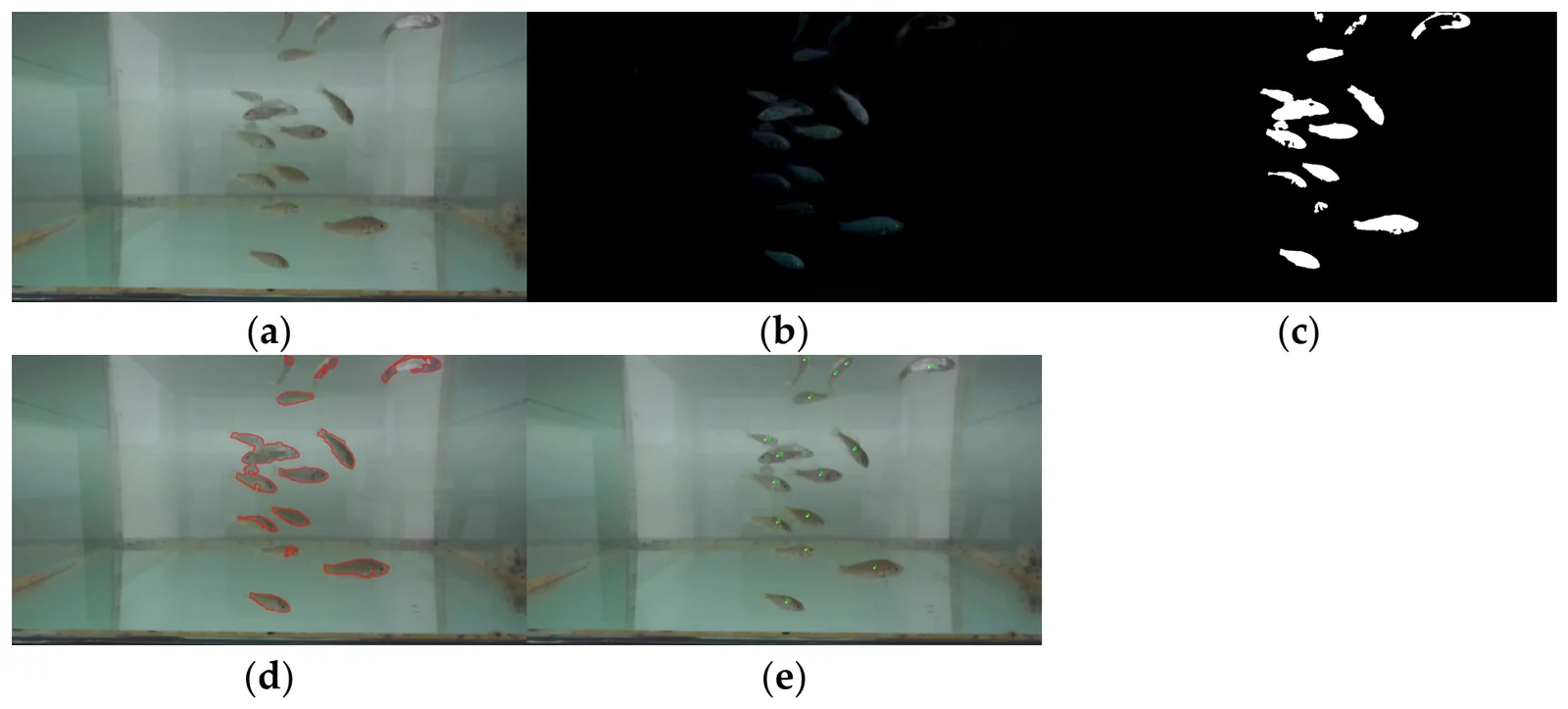
Data processing results. (**a**) Original fish image; (**b**) Image differencing result; (**c**) Image enhancement result; (**d**) Target segmentation result, the red contours indicate fish targets; (**e**) Feature point extraction result, the green dots indicate fish feature points.

Following the binocular image processing, all fish images in the dataset were processed to segment the fish targets and obtain their feature points, paving the way for the extraction of 2D and 3D spatial features. In Figure 12, subfigures (a)–(e) represent the original fish image, image differencing result, image enhancement result, target segmentation result, and feature point extraction result, respectively.

From Figure 12, it can be observed that, after image processing, fish targets in the images can be accurately segmented and identified, benefiting later feature extraction.

### 3.3. Two-Dimensional Spatial Feature Extraction Results

**Figure 13:**
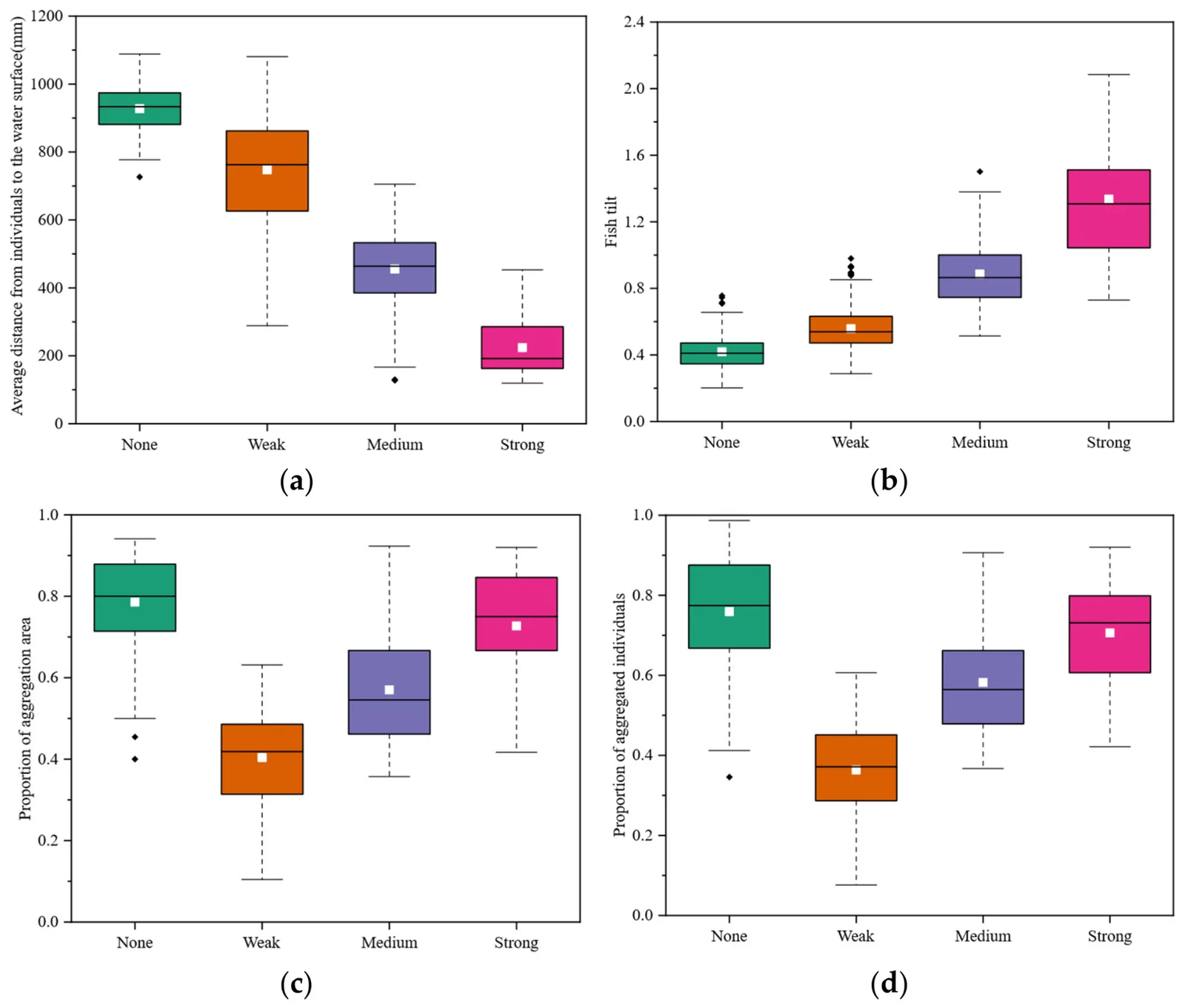
Boxplot of 2D fish spatial features. (**a**) ADS; (**b**) FT; (**c**) PAA; (**d**) PAI. The horizontal line inside the box represents the median, the lower and upper boundaries of the box represent the first and third quartiles, respectively, and the whiskers indicate the data range. The white square represents the mean, and the black points represent outliers. The sample sizes for the none, weak, medium, and strong feeding-intensity levels were 2687, 2562, 2159, and 2095 image frames, respectively.

Based on the accurate segmentation and identification of individual fish, four 2D features, ADS, FT, PAA, and PAI, are extracted. To more intuitively illustrate the differences in spatial features under different feeding intensities, box plots of these features are constructed, as shown in Figure 13.

From Figure 13, it can be seen that the four extracted 2D behavioural features show distinct distributional differences across feeding intensities. Taking feature FT in Figure 13b as an example, it can be seen that FT shows a gradual increasing trend as feeding intensity increases. In the non-feeding state, fish stay in the middle and lower water layers and swim horizontally, producing the smallest and most concentrated FT values. Under the weak feeding state, some fish individuals exhibit a slight tilt associated with feeding behaviour, and FT increases slightly compared with the non-feeding state. Under the medium feeding state, more fish individuals rise towards the water surface and show tilted postures, corresponding to a noticeable increase in FT. Under the strong feeding state, a wider distribution and higher overall FT values are observed, reflecting a greater proportion of tilted fish postures. This phenomenon is consistent with the spatial features of tilted feeding under feeding demand, indicating that FT provides useful information for distinguishing different feeding-intensity levels. The other extracted features also show different distributional patterns across feeding intensities, further indicating their relevance to feeding-intensity assessment.

### 3.4. Three-Dimensional Spatial Feature Extraction Results

#### 3.4.1. Feature Point Matching Results Based on MPR

**Figure 14:**
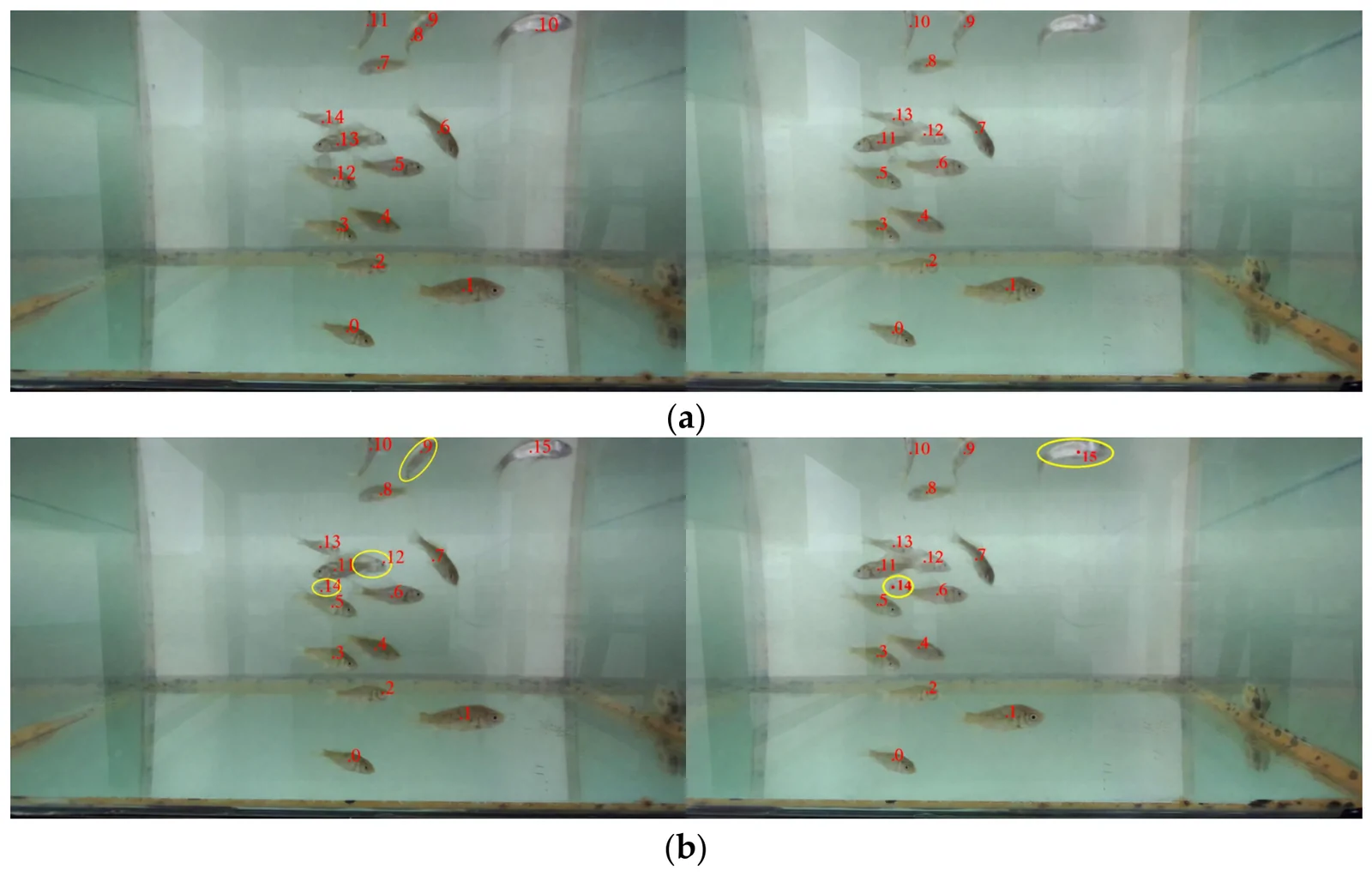
Feature point matching results based on MPR. (**a**) Before using the MPR; (**b**) After using the MPR. The yellow circles highlight the restored feature points, and the red numbers indicate the feature-point labels used for matching.

Accurate feature point matching improves the precision of 3D fish feature acquisition and facilitates accurate assessment of fish feeding intensity. Fish images with missing feature points are selected, and the effects before and after applying the proposed MPR algorithm are compared, as shown in Figure 14.

From Figure 14a, it can be seen that, before applying the proposed MPR algorithm, the number of feature points in the left image is 15, whereas in the right image it is 14. Points 8 and 9 in the left image correspond to the same individual, point 10 is missing in the right image, and the individual corresponding to point 12 in the right image is not detected in the left image due to occlusion. Therefore, missing feature points exist in both the left and right images. This situation not only affects the identification of individuals and prevents proper matching of feature points between binocular images but also hinders the reconstruction of 3D fish features, thereby affecting the performance of feeding intensity assessment. Consequently, the MPR algorithm is required to restore the missing feature points before 3D features can be computed.

The images after restoring feature points using the proposed MPR algorithm are shown in Figure 14b. It can be seen that the MPR algorithm successfully restores the missing feature points in both images. The individual previously labelled as points 8 and 9 in the left image is correctly reassigned. In addition, the feature points missing due to occlusion in the left image are also restored. After restoration, the feature points in the left and right images are matched and reordered, as indicated by the numbering in Figure 14b.

From the comparison before and after applying the MPR algorithm, the example shown in Figure 14 demonstrates that the proposed MPR-based method can restore missing feature points and facilitate subsequent feature-point matching, which is crucial for the subsequent computation of 3D fish features and feeding intensity assessment.

To further provide a direct quantitative evaluation of the restoration performance of MPR, the missing-feature-point records obtained during actual binocular image processing were further analysed. A total of 798 missing feature points were recorded, of which 762 were successfully restored by MPR, corresponding to a restoration rate of 95.49%. Among the successfully restored feature points, 741 were correctly matched with their corresponding fish, resulting in a matching accuracy of 97.24%. These results provide a direct quantitative evaluation of the ability of MPR to restore and match missing fish feature points.

#### 3.4.2. Three-Dimensional Feature Calculation Results Based on WDT

**Figure 15:**
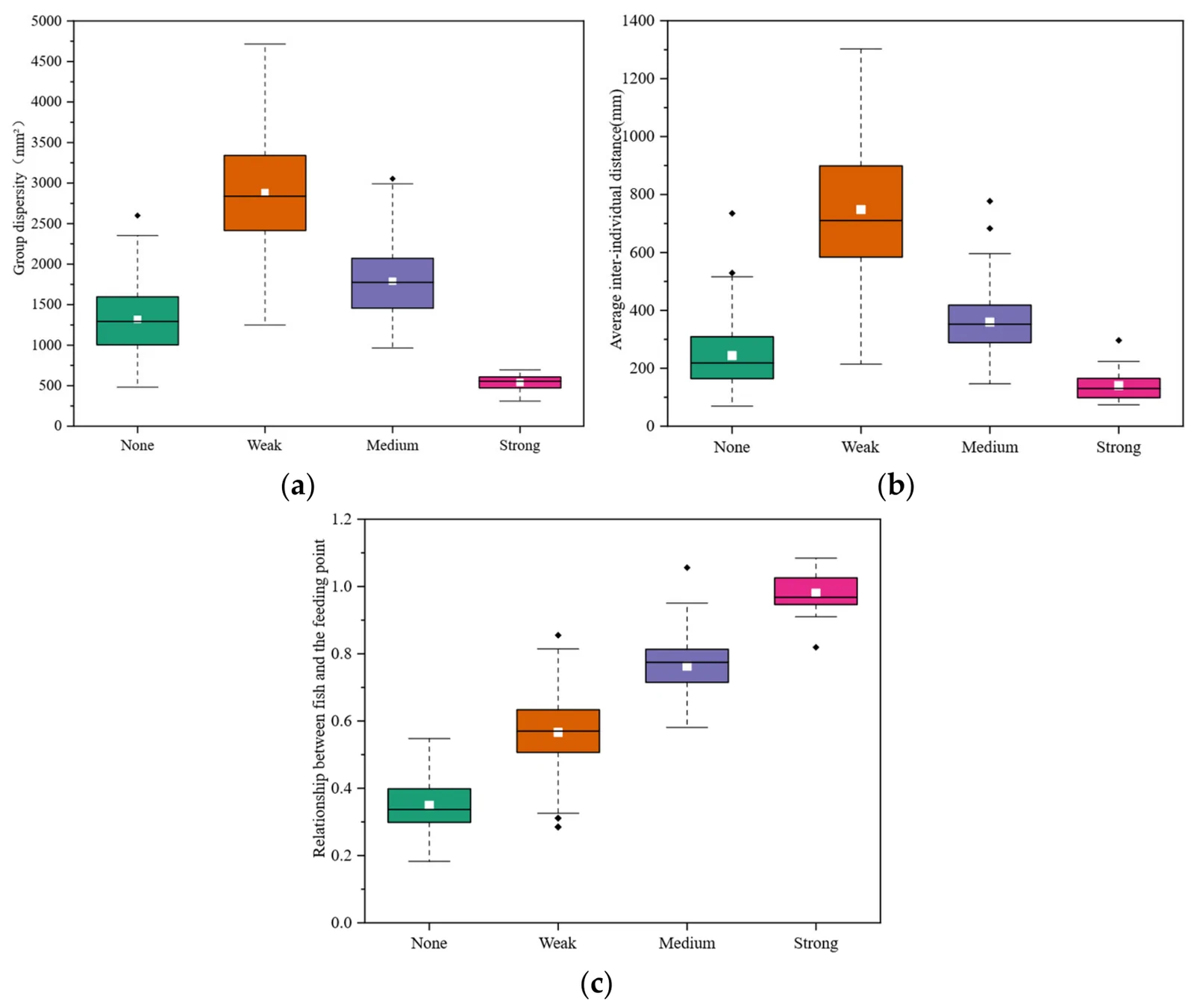
Boxplot of 3D fish spatial features. (**a**) GD; (**b**) AID; (**c**) RFPR. The sample sizes for the none, weak, medium, and strong feeding-intensity levels were 2687, 2562, 2159, and 2095 image frames, respectively.

After restoring the missing feature points using the MPR algorithm, the 3D coordinates of fish feature points are reconstructed based on the principles of binocular vision. On this basis, the WDT algorithm is employed to accurately obtain three 3D features of GD, AID, and RFPR. The extraction results of these 3D features are shown in Figure 15.

Figure 15 shows that the three extracted 3D features exhibit distinct distributional differences across feeding intensities. Taking feature GD in Figure 15a as an example, in the non-feeding state, fish tend to congregate close to the bottom, resulting in relatively low values of GD. Under the weak feeding state, some fish individuals rise towards the water surface while others remain in the lower water layers, corresponding to a more dispersed distribution of the fish school and higher values of GD with larger fluctuations and a wider range. Under the medium feeding state, the spatial dispersity of the fish school decreases, with GD showing lower values than under the weak feeding state. Under the strong feeding state, a more aggregated spatial distribution is observed, with GD showing relatively low values and a narrow distribution range. In summary, the extracted 3D features exhibit different distributional patterns across feeding-intensity levels, further indicating that these features provide useful information for feeding-intensity assessment.

To further examine the differences in the extracted spatial features among feeding-intensity levels, a Kruskal–Wallis non-parametric test was performed for the seven spatial features. For this auxiliary statistical comparison, a balanced subset of 912 frame-level samples was used, comprising 228 samples from each of the four feeding-intensity levels. Equal group sizes were adopted to maintain balanced representation across the feeding-intensity levels. The *H* statistic, *p* value, and effect size (*ε^2^*) were reported for each spatial feature. Under the current frame-level analysis, differences were observed among the four feeding-intensity groups for all seven spatial features (all *p* < 0.001). The detailed statistical results are presented in Table 4.

### 3.5. Fish Feeding Intensity Assessment Results Based on TabPFN

**Figure 16:**
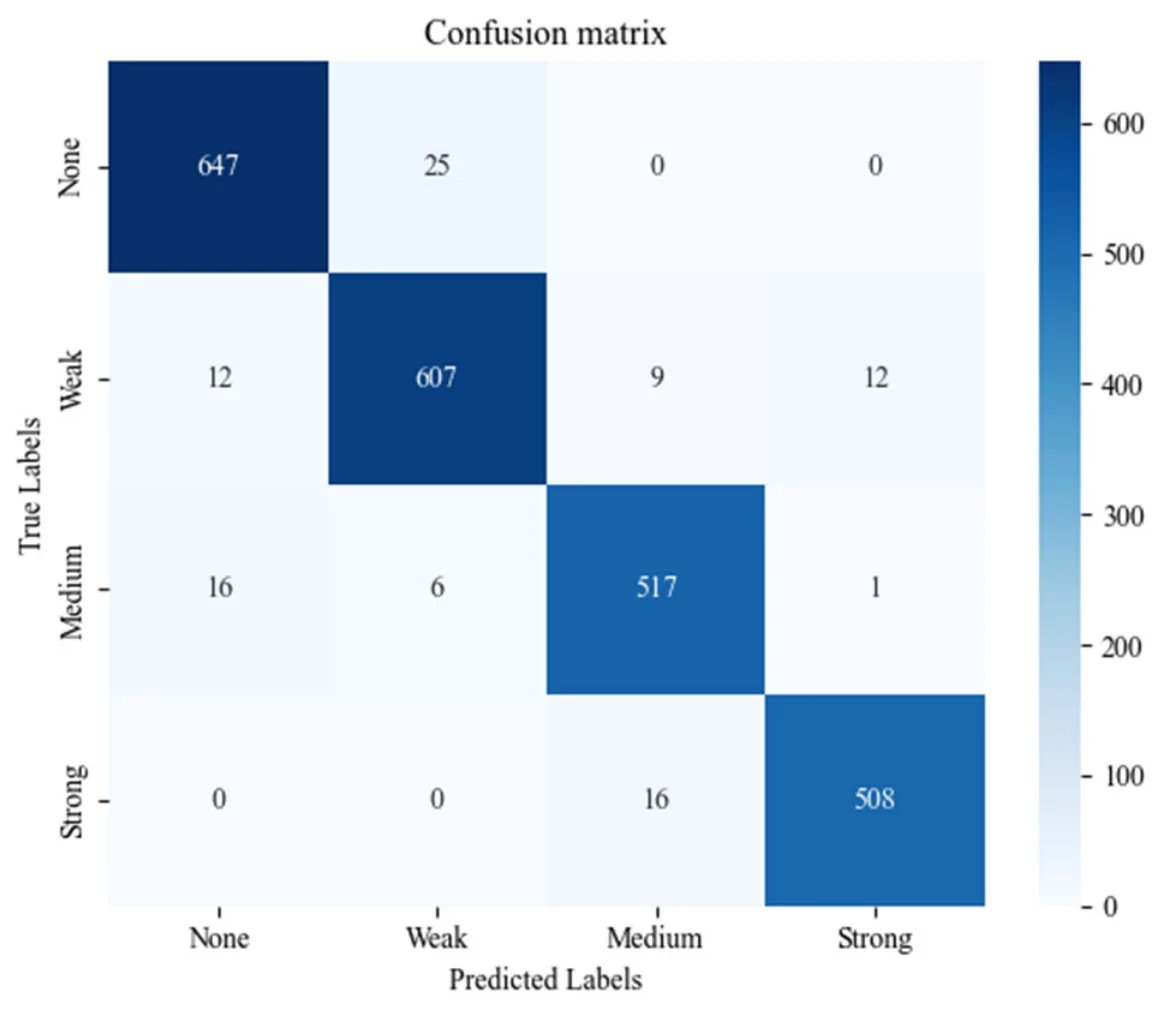
Confusion matrix of fish feeding intensity assessment results based on TabPFN.

This study proposes a TabPFN-based method for fish feeding intensity assessment, classifying feeding intensity into four levels of none, weak, medium, and strong. The corresponding confusion matrix is presented in Figure 16, and the detailed class-wise confusion matrix counts are provided in Table 5.

The overall accuracy based on the TabPFN model reaches 95.92%, the average F1- score is 95.96%, and the balanced accuracy is 95.95%. The precision, recall, specificity, and F1-score for each intensity category are presented in Table 6.

According to Table 6, the proposed method achieves high classification performance across all four metrics, with all values exceeding 94% for each feeding intensity. Specifically, the specificity of the strong category reaches the highest value of 99.30%, while the strong intensity category achieves the highest precision (97.50%), recall (96.95%), and F1-score (97.22%). All overall averages remain above 95.90%, indicating that our proposed model has well-balanced classification capability.

Across categories, strong feeding shows consistently higher metrics, a result of its more distinctive spatial features. For example, during strong feeding, fish collectively rise to the surface and exhibit pronounced aggregation, leading to substantially reduced ADS and GD relative to other intensity levels. Although the weak category has a relatively lower precision, recall and F1-score, its specificity reaches 98.21%, indicating that the model demonstrates high reliability in distinguishing weak feeding from other categories, with a very low misclassification rate.

To further evaluate the performance stability of the proposed method under different data partitions, five repeated experiments were conducted, with the data repartitioned and the model independently trained and tested in each experiment. The corresponding results are presented in Table 7.

Across the five repeated experiments, all evaluation metrics remained at relatively high levels. The standard deviations ranged from 0.31% to 0.99%, further indicating the performance stability of the model.

In addition to the five repeated experiments, three complete feeding videos that were entirely independent of the original data were used to evaluate the established model on unseen feeding events. None of the frames extracted from these videos were involved in model training, validation, testing, model selection, or parameter adjustment. The additional videos were processed using the same procedure as the original dataset. After preprocessing, a total of 822 samples were obtained. The sample distribution of the three independent feeding videos is presented in Table 8.

The established model was directly applied to these samples for prediction without any additional training or parameter adjustment. The overall accuracy on the independent test set was 95.99%, and the class-wise evaluation results are presented in Table 9.

These results were comparable to those obtained on the original test set, further indicating good consistency in the model’s assessment performance on independent feeding events.

### 3.6. Ablation Experiment Results

To validate the effectiveness of the proposed fish feeding intensity assessment method, some ablation experiments are conducted, and the results are displayed in Table 10.

From Table 10, it can be observed that the proposed method achieves high values across the evaluated metrics, with an accuracy of 95.92%, precision of 95.97%, recall of 95.95%, specificity of 98.63%, and F1-score of 95.96%. Compared with TabPFN-MPR, the proposed method shows higher values across the evaluated metrics. By employing the WDT-based method to compute 3D features for feeding intensity assessment, the proposed method achieves the aforementioned performance. In contrast, the TabPFN-MPR method without WDT-based 3D feature extraction yields an accuracy of 94.78%, precision of 92.40%, recall of 93.05%, specificity of 96.62%, and F1-score of 92.73%, indicating a noticeable decline across all evaluation metrics. This demonstrates the importance of the WDT-based 3D feature computation method in the feeding intensity assessment task. Furthermore, the MPR-based feature point matching method proposed in this study also plays a crucial role in the assessment process. When WDT was retained but MPR was removed, the accuracy decreased from 95.92% to 93.20%, while the F1-score decreased from 95.96% to 91.34%. Overall, both the MPR-based feature point matching method and the WDT-based 3D feature computation method contribute to improving the accuracy of fish feeding intensity assessment, validating the effectiveness of the proposed approach.

To further evaluate the stability and statistical reliability of the ablation results, five repeated experiments were conducted for each ablation model, and the results are presented in Table 11.

The repeated experiments showed an overall trend consistent with the original ablation study. The proposed method achieved the highest mean accuracy and average F1-score across the five repeated experiments, whereas removing either the MPR module or the WDT module resulted in decreased model performance, further indicating that both modules consistently contributed to improving model performance.

### 3.7. Comparison Results

#### 3.7.1. Comparison Results Based on 2D and 3D Spatial Features

**Figure 17:**
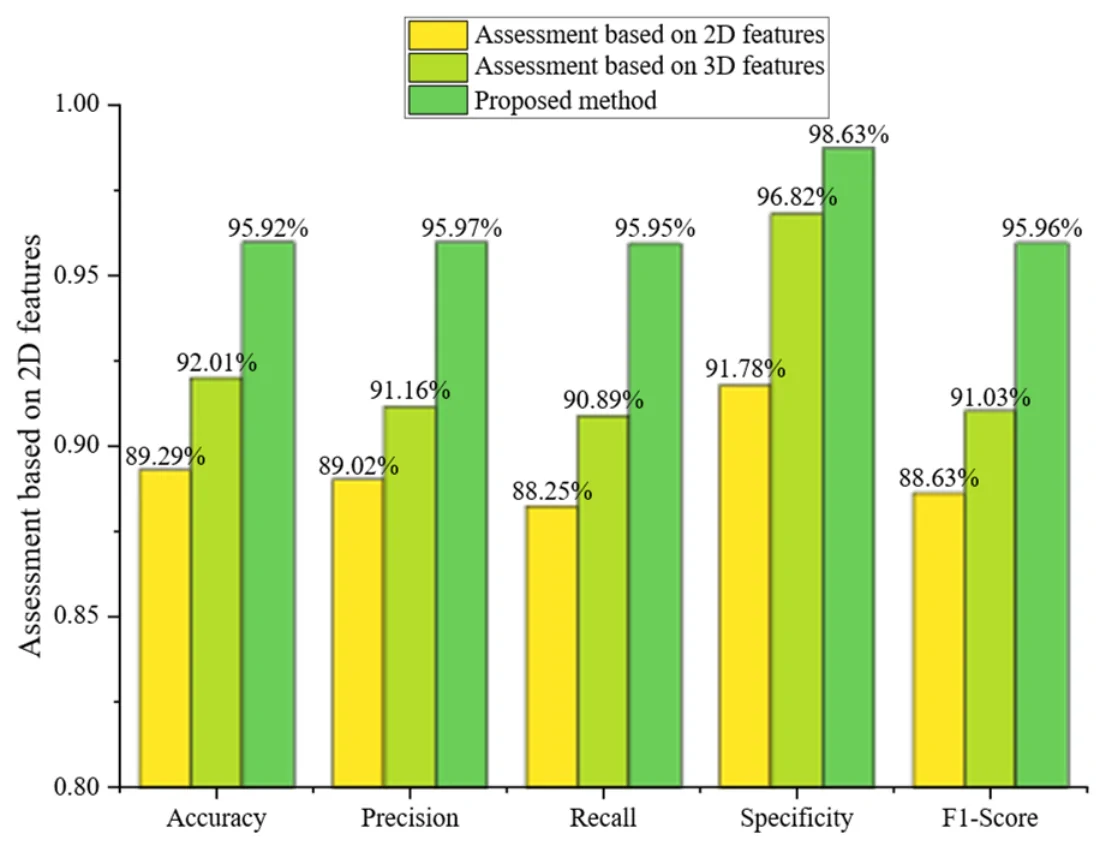
Comparison result based on 2D and 3D features.

In the proposed method, missing feature points are restored to reconstruct the 3D spatial feature points of fish, and multiple 3D spatial features are extracted based on the WDT algorithm for fish feeding intensity assessment. To compare the performance of 2D and 3D spatial features, fish feeding intensity assessment is conducted separately using four 2D features and three 3D features, and the results are compared with those of the proposed method. The comparison results are presented in Figure 17 and Table 12.

As shown in Figure 17, the proposed method achieves an accuracy of 95.92%, precision of 95.97%, recall of 95.95%, specificity of 98.63%, and an F1-score of 95.96%. In contrast, the method based on 2D features yields an accuracy of 89.29%, precision of 89.02%, recall of 88.25%, specificity of 91.78%, and F1-score of 88.63%, representing relative improvements of 7.43%, 7.81%, 8.73%, 7.46%, and 8.27%, respectively, for the proposed method. The method based on 3D features achieves an accuracy of 92.01%, precision of 91.16%, recall of 90.89%, specificity of 96.82%, and F1-score of 91.03%. In comparison, the proposed method achieves relative improvements of 4.25%, 5.28%, 5.57%, 1.87%, and 5.42%, respectively. Moreover, the assessment method based on 3D features achieved higher observed values than the method based on 2D features across all evaluation metrics, indicating that the extracted 3D features are more effective in describing fish feeding behaviour and reflecting feeding intensity. These results demonstrate the complementary nature of the 2D and 3D spatial features and support the research hypothesis that their fusion can improve feeding intensity assessment performance compared with using either type of feature alone.

#### 3.7.2. Comparison Results Based on Different Triangulation Algorithms

**Figure 18:**
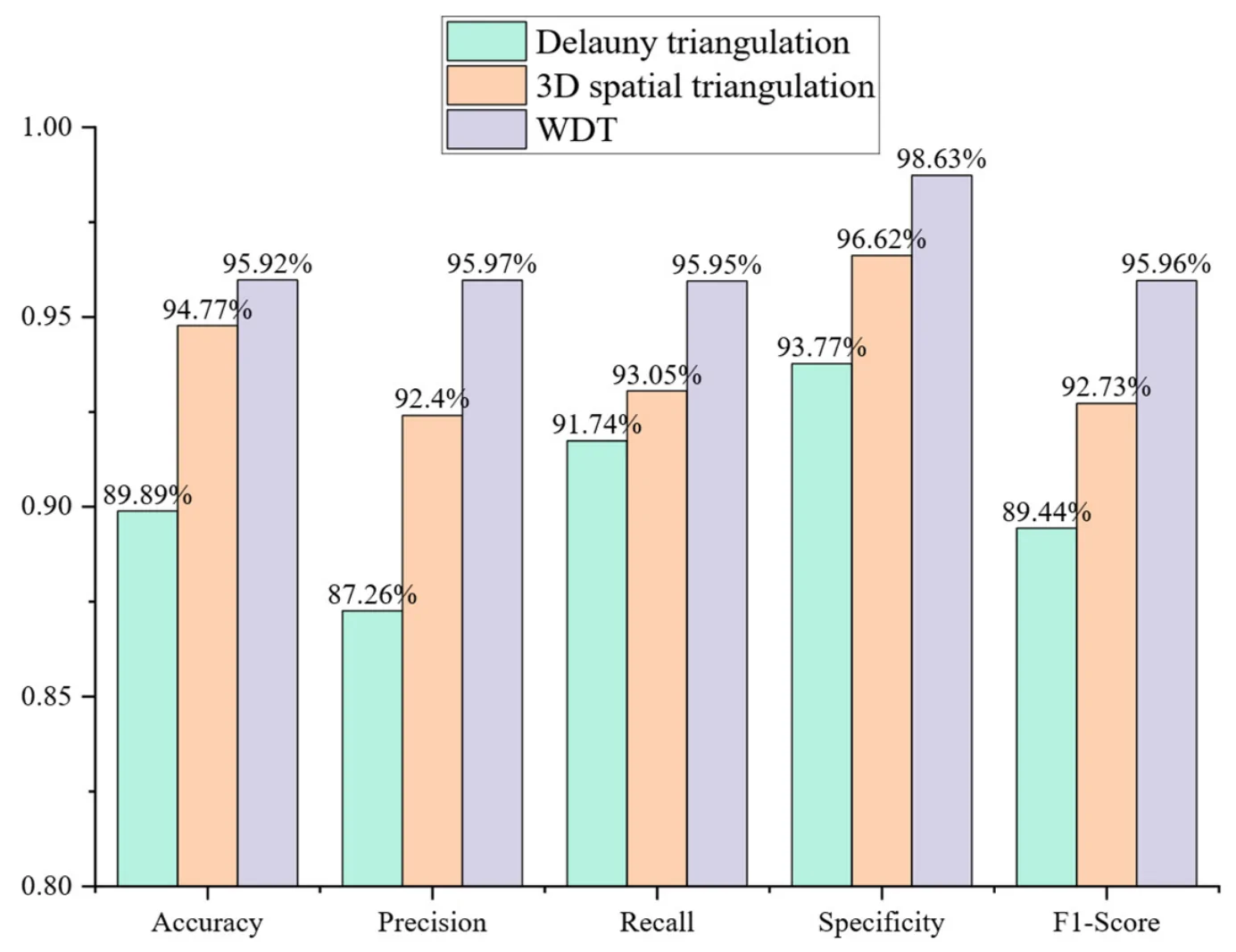
Performance comparison of different triangulation algorithms.

In the study, a WDT-based 3D feature computation approach is proposed. To further evaluate the effectiveness of WDT in 3D feature extraction and fish feeding intensity assessment, it is compared with Delaunay triangulation [29] and 3D spatial triangulation [30]. The visual comparison results are shown in Figure 18.

From Figure 18, it can be observed that, for fish feeding intensity assessment, the WDT-based method achieves an accuracy of 95.92%, representing relative improvements of 6.71% and 1.21% over Delaunay triangulation at 89.89% and 3D spatial triangulation at 94.77%, respectively. The precision reaches 95.97%, representing relative improvements of 9.98% and 3.86% over Delaunay triangulation at 87.26% and 3D spatial triangulation at 92.40%, respectively. The recall was 95.95%, representing relative improvements of 4.59% and 3.12% compared to Delaunay triangulation at 91.74% and 3D spatial triangulation at 93.05%, respectively. Specificity was 98.63%, representing relative improvements of 5.18% and 2.08% over Delaunay triangulation at 93.77% and 3D spatial triangulation at 96.62%, respectively. The F1-score was 95.96%. As shown in Figure 18, the proposed WDT-based 3D feature extraction method achieved higher observed values than the comparison methods across all evaluation metrics, indicating that it can accurately obtain 3D fish features and improve the accuracy of fish feeding intensity assessment. Under the current evaluation setting, these comparative results provide empirical support for the use of the proposed WDT-based weighting strategy, although they do not establish that the current weighting function is optimal.

#### 3.7.3. Comparison Results Based on Different Classification Models

The performance of TabPFN is evaluated by comparing it with several alternative feeding intensity assessment models, including TabNet [31], XGBoost [32], LGBM [33], RF [34], MLP [35], DT [36], and 1D-CNN [37]. Among these models, the 1D-CNN was included as a neural-network baseline to provide a further empirical reference for the performance comparison among different classification models. The comparison results are presented in Table 13.

As shown in Table 13, under the current evaluation setting, the proposed TabPFN method achieved the highest observed values across all five evaluation metrics, with an accuracy of 95.92% and an F1-score of 95.96%. Among the comparison models, MLP, DT, and TabNet achieved relatively higher accuracies of 93.02%, 92.94%, and 92.65%, respectively. In contrast, the 1D-CNN achieved an accuracy of 78.19% and an F1-score of 70.36%, with lower classification performance on the current seven-dimensional spatial features than most of the other comparison models.

**Figure 19:**
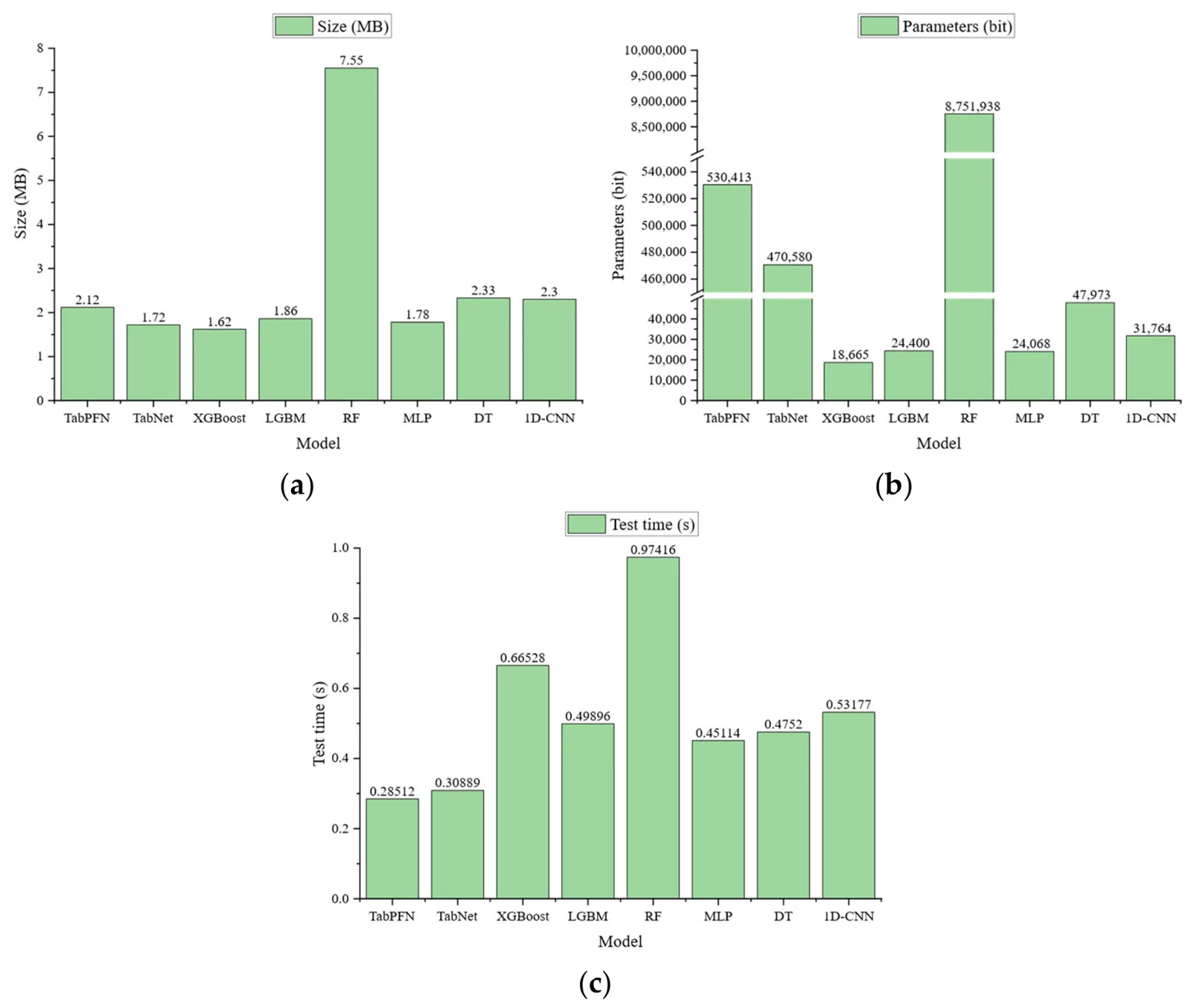
Comparison results of parameters in the feeding intensity assessment model. (**a**) Model size (MB); (**b**) Parameters (bit); (**c**) Test time (s).

In addition, to provide a comprehensive comparison of different classification models, indicators such as model size and the number of parameters were also evaluated, and the corresponding results are presented in Figure 19.

Figure 19 demonstrates that TabPFN has a relatively small model size of 2.12 MB, which provides computational advantages for future practical deployment. The model size was calculated based on 32-bit floating-point parameters, without quantisation or compression. When evaluated on 2376 test samples, the method requires only 0.28512 s in total and 0.00012 s per sample, indicating a short test time under the current experimental environment.

Therefore, considering model accuracy, model size, and computational efficiency, the TabPFN model showed favourable overall performance under the current experimental setting. Further validation under more diverse aquaculture conditions is required before practical deployment.

#### 3.7.4. Comparison Results with Other Methods

To further validate the effectiveness of the proposed binocular vision-based assessment method for fish feeding intensity via 2D and 3D spatial feature fusion, it is compared with other existing methods, including those proposed by Yu et al. [38], Feng et al. [13], Zhang et al. [39] and Zhang et al. [20]. Zhang et al. [20] is our previous work on assessing fish feeding intensity based on 2D spatial features. For a fair comparison, all comparative methods were reimplemented and evaluated on the dataset used in the present study under the same data partition and evaluation metrics. The comparison results are presented in Table 14.

Table 14 presents the comparison results between the proposed method and the methods of Yu et al. [38], Feng et al. [13], Zhang et al. [39] and Zhang et al. [20]. As shown in Table 14, the proposed method achieved an accuracy of 95.92% and an F1-score of 95.96%. The accuracies reported by the comparative methods ranged from 78.15% to 95.96%, while their F1-scores ranged from 77.65% to 93.38%. The proposed method achieved higher accuracy than the other deep learning-based and image processing-based comparison methods, while achieving an accuracy comparable to that of our previous 2D spatial feature-based method. Meanwhile, the proposed method achieved higher Precision, Recall, Specificity, and F1-score than the previous method. This indicates that incorporating 3D spatial features from binocular vision better captures the characteristics of different feeding intensities, thereby improving assessment accuracy.

**Figure 20:**
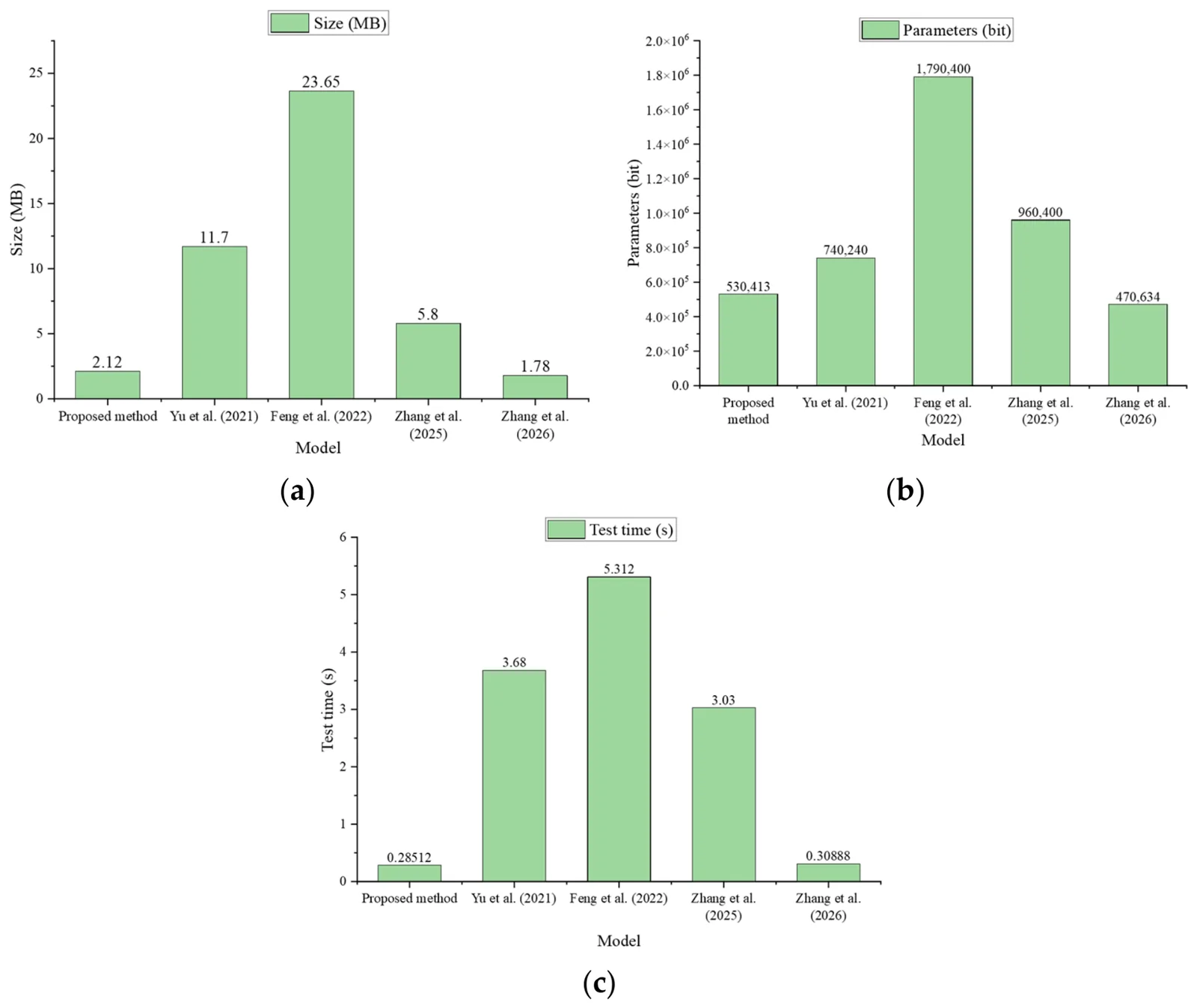
Comparison results of parameters in different feeding intensity assessment models. (**a**) Model size (MB); (**b**) Parameters (bit); (**c**) Test time (s). The comparative methods correspond to Yu et al. [38], Feng et al. [13], Zhang et al. [39], and Zhang et al. [20].

In addition, model parameters of different assessment methods are further compared, and the corresponding results are presented in Figure 20.

As shown in Figure 20, the model size of the proposed method is only 2.12 MB, which is much smaller than 11.7 MB, 23.65 MB, and 5.8 MB of the comparative methods. The proposed method has 530,413 parameters. Consequently, the proposed method shows favourable results compared with the comparative methods in terms of model size, portability, and computational efficiency for feeding intensity assessment. As shown in Figure 20, the proposed model has a size of 2.12 MB and 530,413 parameters, both of which are lower than those reported by Yu et al. [38], Feng et al. [13] and Zhang et al. [39], and slightly higher than those in our previous study on fish feeding intensity assessment using 2D spatial features [20]. Its test time for 2376 images is just 0.2851 s, which is also shorter than that of all the comparison methods. Therefore, taking into account model size, the number of parameters, computational efficiency and the accuracy of feeding intensity assessment, the proposed method showed favourable overall performance under the current evaluation setting.

## 4. Discussion

### 4.1. Novelty and Advantages of the Proposed Method

Accurately assessing fish feeding intensity is of great significance for understanding their feeding demand, dynamically adjusting feeding strategies, and improving aquaculture efficiency. Given that changes in fish behaviour are accompanied by changes in spatial distribution and structure, a method for assessing fish feeding intensity that integrates 2D and 3D spatial features, utilising binocular vision technology is proposed in this paper. By characterising observable changes in fish spatial distribution and structure, this study extracts spatially meaningful features that reflect feeding-related spatial variation. It addresses two key limitations of existing methods. Firstly, it overcomes the difficulty of accurately representing variations in feeding intensity when relying solely on surface features such as texture, and secondly, it avoids the ‘black-box’ issue associated with existing methods that use deep learning to automatically extract features from fish images. As we know, although deep learning methods are capable of automatically extracting features from fish images and yielding satisfactory evaluation results, it is difficult to understand the meaning of the extracted features. This means that whilst we are aware of changes in feeding intensity, we do not know why these changes have occurred, resulting in feeding decisions that lack interpretability and credibility. In this paper, we extract four 2D spatial features (ADS, FT, PAA and PAI) and three 3D spatial features (GD, AID and RFPR) to serve as inputs for the TabPFN model in assessing feeding intensity. In our previous study [20], feature-importance analysis based on TabNet-DFWL showed that the seven spatial features were ranked in descending order of importance as GD, ADS, FT, AID, RFPR, PAA, and PAI, providing a further reference for understanding the relative contributions of different behaviour-related spatial features. The experimental results indicate that the proposed approach can effectively assess fish feeding intensity under the controlled conditions evaluated in this study. In addition, the relatively small model size and short test time indicate its computational efficiency and potential for future practical applications. However, further validation under more diverse aquaculture conditions is still required.

### 4.2. Key Distinctions and Advancements from Prior Study

A paper on a method for assessing fish feeding intensity based on spatial features was published previously by us [20]. The published paper utilised monocular vision to extract seven spatial features and employed an improved TabNet classification model to assess fish feeding intensity, achieving an accuracy of 95.96%, precision of 93.44%, recall of 93.33%, specificity of 98.15% and F1-score of 93.38%. The work presented in this paper builds upon our previously published research. Although both studies assess fish feeding intensity from the perspective of spatial features and emphasise model interpretability, the published research relies on monocular vision to extract spatial features, all of which are 2D spatial features, whereas this paper introduces binocular vision technology, emphasising the assessment of fish feeding intensity through the fusion of 2D and 3D spatial features. The two approaches differ fundamentally in terms of the dimensionality of the feature space. Among the seven 2D spatial features extracted in our previous study, group dispersity (GD), average inter-individual distance (AID), and the relationship between fish and the feeding point (RFPR) mainly characterise the spatial positional relationships among fish individuals or between fish and the feeding point, and thus can be naturally extended to three-dimensional space when depth information is available. In contrast, average distance from individuals to the water surface (ADS), fish tilt (FT), proportion of aggregation area (PAA), and proportion of aggregated individuals (PAI) are mainly defined based on vertical position, posture, or two-dimensional projected regions in the side-view image and do not have corresponding three-dimensional forms with clear physical meaning. Therefore, only the 2D forms of these four features are retained in this study. Furthermore, for the remaining three 2D features, we have computed their representations in 3D space, thereby deriving 3D spatial features.

The method for calculating 3D spatial features, which constitutes the main contributions of this paper, comprises two aspects. Firstly, when calculating 3D spatial features, we must match the feature points of the fish in the left and right images under binocular vision. However, as individual fish exhibit significant overlapping and occlusion during feeding, some feature points appear only in the left or right image, making it impossible to match the feature points between the two images. Consequently, a method called MPR that utilises the disparity in the binocular images to restore missing feature points is proposed in the paper, thereby laying the foundation for precise feature point matching. Secondly, to accurately compute the 3D spatial features, a WDT algorithm is proposed. It performs spatial triangulation by treating the spatial feature points as vertices of Delaunay triangles, and assigns specific weights to the triangles based on their participation in the triangular mesh. This enables the data obtained after triangulation to better characterise the spatial organisation of the fish school, thereby supporting subsequent feeding-intensity assessment.

In this study, we also conducted comparative experiments to assess feeding intensity using only 2D spatial features and only 3D spatial features, respectively. We found that the method combining 2D and 3D spatial features achieved better performance, with an accuracy of 95.92%, precision of 95.97%, recall of 95.95%, specificity of 98.63% and F1-score of 95.96%, indicating that 2D and 3D spatial features provide complementary information for feeding-intensity assessment.

### 4.3. Limitations and Future Work

Regarding the experimental setting, although the proposed method has achieved good results on the real fish feeding dataset, it should be noted that the dataset used in this study was acquired under controlled laboratory conditions, where water quality and lighting conditions were relatively stable. In addition, the experiments were conducted using a single fish species in a single experimental tank. Meanwhile, changes in feeding location, feeding amount, stocking density, tank geometry, or camera placement may also affect fish spatial distributions and the resulting feature values. Therefore, the present results are mainly applicable to the experimental conditions evaluated in this study, and further validation under different fish populations and species, aquaculture systems, environmental conditions, and feeding schemes is required to evaluate the applicability of the proposed method.

Regarding methodological aspects, the proposed method employs traditional image processing methods for fish body segmentation and spatial feature extraction, which are relatively sensitive to environmental changes. When there are significant changes in water turbidity or light intensity within the aquaculture environment, the performance of this method may be affected to some extent. To improve the adaptability of the method under more complex environmental conditions, future research will adopt deep learning methods for fish body segmentation to enhance the robustness of the algorithm in complex environments. Compared to traditional image processing methods, deep learning models demonstrate greater adaptability to noise, occlusion and changes in lighting conditions. Data augmentation strategies will also be introduced to further improve the model’s generalisation ability across different aquaculture scenarios. Regarding the WDT weighting mechanism, the current weighting function was designed based on the local topological structure of the tetrahedral mesh, and its present validation is mainly based on downstream feeding-intensity assessment performance. Future studies will compare alternative weighting functions and parameterisations under different experimental conditions to further evaluate the robustness and appropriateness of the weighting strategy. Collectively, these improvements represent key steps in advancing the method from laboratory settings to practical applications such as industrial recirculating aquaculture systems and pond farming.

Regarding the representation of feeding intensity, the present study classifies fish feeding intensity into four discrete levels: none, weak, medium, and strong. Although this classification can distinguish typical feeding states, it cannot fully characterise the continuous temporal variation in feeding intensity. Future work will therefore explore finer-grained and continuous quantification methods to provide a more detailed representation of the dynamic changes in fish feeding intensity over time.

## 5. Conclusions

To address the limitation of incomplete behavioural representation caused by monocular vision, which relies solely on 2D features and restricts the accuracy of feeding intensity assessment, a binocular vision-based assessment method for fish feeding intensity via 2D and 3D spatial feature fusion is proposed. This method enables the accurate extraction of both 2D and 3D spatial features of fish, effectively overcoming the limitation of insufficient representation of vertical behaviours in 2D images and compensating for errors caused by the absence of depth information, thereby improving the accuracy of feeding intensity assessment. An MPR method is introduced to address the issue of missing feature points caused by occlusion and overlap among individuals in binocular images, facilitating the restoration and subsequent matching of fish feature points. In addition, a WDT algorithm is proposed, which effectively enhances the representation of fish spatial dispersity and improves the accuracy of feeding intensity assessment.

Under the experimental conditions evaluated in this study, the proposed method achieved an accuracy of 95.92%, and the fusion of 2D and 3D spatial features showed better observed performance than using either type of spatial feature alone. These results demonstrate the potential of combining 2D and 3D spatial information for fish feeding-intensity assessment. However, the present findings are limited to juvenile Asian crucian carp in a single experimental tank under relatively controlled conditions. Further validation involving different fish populations and species, aquaculture systems, water turbidity and illumination conditions, and independent feeding events is required to further evaluate the robustness and generalisability of the proposed method.

## Figures and Tables

**Table 1:** Sample classification criteria of the dataset.

Feeding Intensity	Description
None	Fish show no response to the supplied food.
Weak	Fish consume only food that falls directly in front of them and show little active movement toward the food.
Medium	Fish move to take food but tend to return to their original positions after feeding.
Strong	Fish move actively among food items and continuously consume the food.

**Table 2:** Dataset partitioning details.

Feeding Intensity	Training Set	Validation Set	Test Set	Total
None	1613	402	672	2687
Weak	1536	386	640	2562
Medium	1296	323	540	2159
Strong	1257	314	524	2095
Total	5702	1425	2376	9503

**Table 3:** Model configuration and hyperparameter settings.

Model	Parameter Name	Parameter Value
TabPFN	Version	6.4.0
	Parameter update	Frozen
	Early stopping	Not applicable
TabNet	max_epochs	500
	batch_size	1024
	learning_rate	0.001
XGBoost	n_estimators	500
	learning_rate	0.001
	max_depth	−1
	subsample	0.5
LGBM	num_leaves	31
	max_depth	−1
	n_estimators	500
	num_threads	0
	learning_rate	0.001
RF	n_estimators	500
	max_depth	None
	min_samples_split	2
	min_samples_leaf	1
MLP	input_size	7
	hidden_size	2
	output_size	4
	hidden_dim	128
DT	max_depth	None
	min_samples_split	2
	min_samples_leaf	1
	criterion	gini
1D-CNN	num_of_Convolution	2
	in_channels_1	1
	out_channels_1	16
	in_channels_2	16
	out_channels_2	32
	kernel_size	3
	max_epochs	500
	batch_size	1024
	learning_rate	0.001

**Table 4:** Kruskal–Wallis test results for the extracted spatial features across different feeding-intensity levels.

Feature	*H* Statistic	*p* Value	Effect Size (*ε*^2^)
GD	677.93	<0.001	0.743
ADS	550.1	<0.001	0.603
PAA	474.47	<0.001	0.519
PAI	515.76	<0.001	0.565
AID	539.87	<0.001	0.591
FT	535.95	<0.001	0.587
RFPR	548.1	<0.001	0.600

*H* denotes the Kruskal–Wallis test statistic; *p* denotes the *p*-value; *ε*^2^ denotes the effect size.

**Table 5:** Classification results of fish feeding intensity assessment based on TabPFN.

True Class	Predicted None	Predicted Weak	Predicted Medium	Predicted Strong
None	647	25	0	0
Weak	12	607	9	12
Medium	16	6	517	1
Strong	0	0	16	508

**Table 6:** Evaluation results of the proposed method on each intensity category.

Feeding Intensity	Precision (%) ↑	Recall (%) ↑	Specificity (%) ↑	F1-Score (%) ↑
None	95.85	96.28	98.36	96.06
Weak	95.14	94.84	98.21	94.99
Medium	95.39	95.74	98.64	95.56
Strong	97.50	96.95	99.30	97.22
Average	95.97	95.95	98.63	95.96

The symbol “↑” in the table indicates that the higher the value of this metric is, the better the performance of the model will be.

**Table 7:** Performance of the proposed method over five repeated experiments.

Metric	Mean ± SD (%)	95% CI (%)
Accuracy	95.24 ± 0.93	94.09–96.40
Average Precision	95.33 ± 0.99	94.10–96.57
Average Recall	95.23 ± 0.91	94.09–96.36
Average Specificity	98.40 ± 0.31	98.02–98.78
Average F1-score	95.26 ± 0.95	94.08–96.44

Values are reported as mean ± standard deviation (SD) over five repeated experiments; CI denotes confidence interval.

**Table 8:** Sample distribution of the three independent feeding videos.

Video	Total Samples	None	Weak	Medium	Strong
Video 1	252	87	50	60	55
Video 2	276	94	54	68	60
Video 3	294	99	58	72	65
Total	822	280	162	200	180

**Table 9:** Evaluation results for each feeding-intensity category in the independent test.

Feeding Intensity	Precision (%) ↑	Recall (%) ↑	Specificity (%) ↑	F1-Score (%) ↑
None	98.55	97.14	99.26	97.84
Weak	90.06	95.06	97.42	92.49
Medium	96.04	97.00	98.71	96.52
Strong	97.69	93.89	99.38	95.75
Average	95.58	95.77	98.69	95.65

**Table 10:** Performance comparison of different ablation configurations.

Assessment Method	MPR	WDT	Accuracy (%) ↑	Average Precision (%) ↑	Average Recall (%) ↑	Average Specificity (%) ↑	Average F1-Score (%) ↑
Proposed method	√	√	95.92	95.97	95.95	98.63	95.96
TabPFN-MPR	√	-	94.78	92.40	93.05	96.62	92.73
TabPFN-WDT	-	√	93.20	91.48	91.22	96.93	91.34
TabPFN	-	-	92.73	91.34	91.17	95.41	91.25

“√” indicates that the corresponding module is included, whereas “-” indicates that it is not included.

**Table 11:** Performance of the ablation models over five repeated experiments.

Assessment Method	MPR	WDT	Accuracy		Average F1-Score	
			Mean ± SD (%)	95% CI (%)	Mean ± SD (%)	95% CI (%)
Proposed method	√	√	95.24 ± 0.93	94.09–96.40	95.26 ± 0.95	94.08–96.44
TabPFN-MPR	√	-	93.83 ± 0.88	92.74–94.92	93.33 ± 0.90	92.21–94.45
TabPFN-WDT	-	√	92.06 ± 0.92	90.91–93.20	91.78 ± 0.97	90.58–92.98
TabPFN	-	-	91.91 ± 0.79	90.92–92.89	91.98 ± 0.80	90.98–92.97

Values are reported as mean ± standard deviation (SD) over five repeated experiments; CI denotes confidence interval.

**Table 12:** Evaluation results based on 2D and 3D spatial features.

Feature Set	Accuracy (%) ↑	Precision (%) ↑	Recall (%) ↑	Specificity (%) ↑	F1-Score (%) ↑
2D features	89.29	89.02	88.25	91.78	88.63
3D features	92.01	91.16	90.89	96.82	91.03
Proposed method	95.92	95.97	95.95	98.63	95.96

**Table 13:** Comparison result of feeding intensity assessment based on different models.

Model	Accuracy (%) ↑	Average Precision (%) ↑	Average Recall (%) ↑	Average Specificity (%) ↑	Average F1-Score (%) ↑
TabPFN	95.92	95.97	95.95	98.63	95.96
TabNet	92.65	91.90	89.27	95.66	90.57
XGBoost	84.60	83.21	75.78	94.04	79.32
LGBM	88.92	85.05	81.85	93.57	83.42
RF	91.33	88.42	85.11	96.48	86.73
MLP	93.02	90.26	88.25	96.15	89.24
DT	92.94	91.03	90.71	97.91	90.87
1D-CNN	78.19	73.37	67.59	90.62	70.36

**Table 14:** Comparison results based on different assessment methods.

Method	Accuracy (%) ↑	Average Precision (%) ↑	Average Recall (%) ↑	Average Specificity (%) ↑	Average F1-Score (%) ↑
Proposed method	95.92	95.97	95.95	98.63	95.96
Yu et al. [38]	83.27	81.14	81.52	92.10	81.33
Feng et al. [13]	78.15	78.30	77.02	89.76	77.65
Zhang et al. [39]	86.21	85.87	84.17	94.05	85.01
Zhang et al. [20]	95.96	93.44	93.33	98.15	93.38

## Data Availability

For data supporting the results of this study, please contact the corresponding author for communication. Due to privacy, these data have not been made public.

## Abbreviations

The following abbreviations are used in this manuscript:

MPR	missing point restoration
WDT	weighted Delaunay triangulation
TabPFN	Tabular Prior-data Fitted Network
ADS	average distance from individuals to the water surface
FT	fish tilt
PAA	proportion of aggregation area
PAI	proportion of aggregated individuals
GD	group dispersity
AID	average inter-individual distance
RFPR	relationship between fish and the feeding point
SGBM	Semi-Global Block Matching
ORB	Oriented FAST and Rotated BRIEF
XGBoost	eXtreme Gradient Boosting
LGBM	Light Gradient Boosting Machine
RF	Random Forest
MLP	Multi-Layer Perceptron
DT	Decision Tree
1D-CNN	1D Convolutional Neural Network
